# Supplementary material for: Met1-specific motifs conserved in OTUB subfamily of green plants enable rice OTUB1 to hydrolyse Met1 ubiquitin chains
Source: Nat Commun. 2022 Aug 9;13:4672. doi: 10.1038/s41467-022-32364-3 (PMC9363410; doi:10.1038/s41467-022-32364-3)
Supplement: Supplementary file 1 — Supplementary Information [file 41467_2022_32364_MOESM1_ESM.pdf]

Supplementary Information: Met1-specific Motifs conserved in OTUB subfamily of green plants enable rice OTUB1 to Hydrolyse Met1 Ubiquitin Chains

L. Lu et al.

Supplementary Table 1 Data Collection and Refinement Statistics

	Apo-OsOTUB1	OsOTUB1~ UbPA	OsOTUB1~M1-diUb-DHA
PDB code	6K9N	6K9P	6KBE
**Data collection**			
Space group	$P2_12_12_1$	$P1$	$P1$
Cell dimensions			
a, b, c (Å)	59.69, 144.81, 155.85	48.90, 50.52, 75.34	57.40, 58.92, 112.40
α, β, γ (°)	90.00, 90.00, 90.00	89.98, 90.01, 87.37	90.06, 89.98, 89.99
Resolution (Å)	47.4-2.25 (2.29-2.25)	41.94-2.047 (2.094-2.047)	41.1-1.98 (2.01-1.98)
R_{merge}	0.097(0.643)	0.137(1.387)	0.055(0.395)
$I / \sigma I$	18.8 (7.5)	16.1 (2.1)	18.6 (4.1)
Completeness (%)	99.7 (99.7)	97.0 (95.8)	96.6 (95.0)
Redundancy	10.8 (10.4)	6.0 (3.3)	3.6 (3.5)
**Refinement**			
Resolution (Å)	2.272 Å	2.047 Å	1.979 Å
No. reflections	58983	41573	94184
R_{work} / R_{free}	16.86 / 22.04	17.47 / 24.47	15.27 / 23.04
No. atoms			
Protein	8724	5276	14722
Ligand/ion	-	-	-
Water	595	571	2053
Average B factors (Å^2)	42.32	47.71	23.122
r.m.s. deviations			
Bond lengths (Å)	0.0181	0.0162	0.0169
Bond angles (°)	1.9609	1.8113	1.9767
Ramachandran outliers	0.3%	0%	0.5%

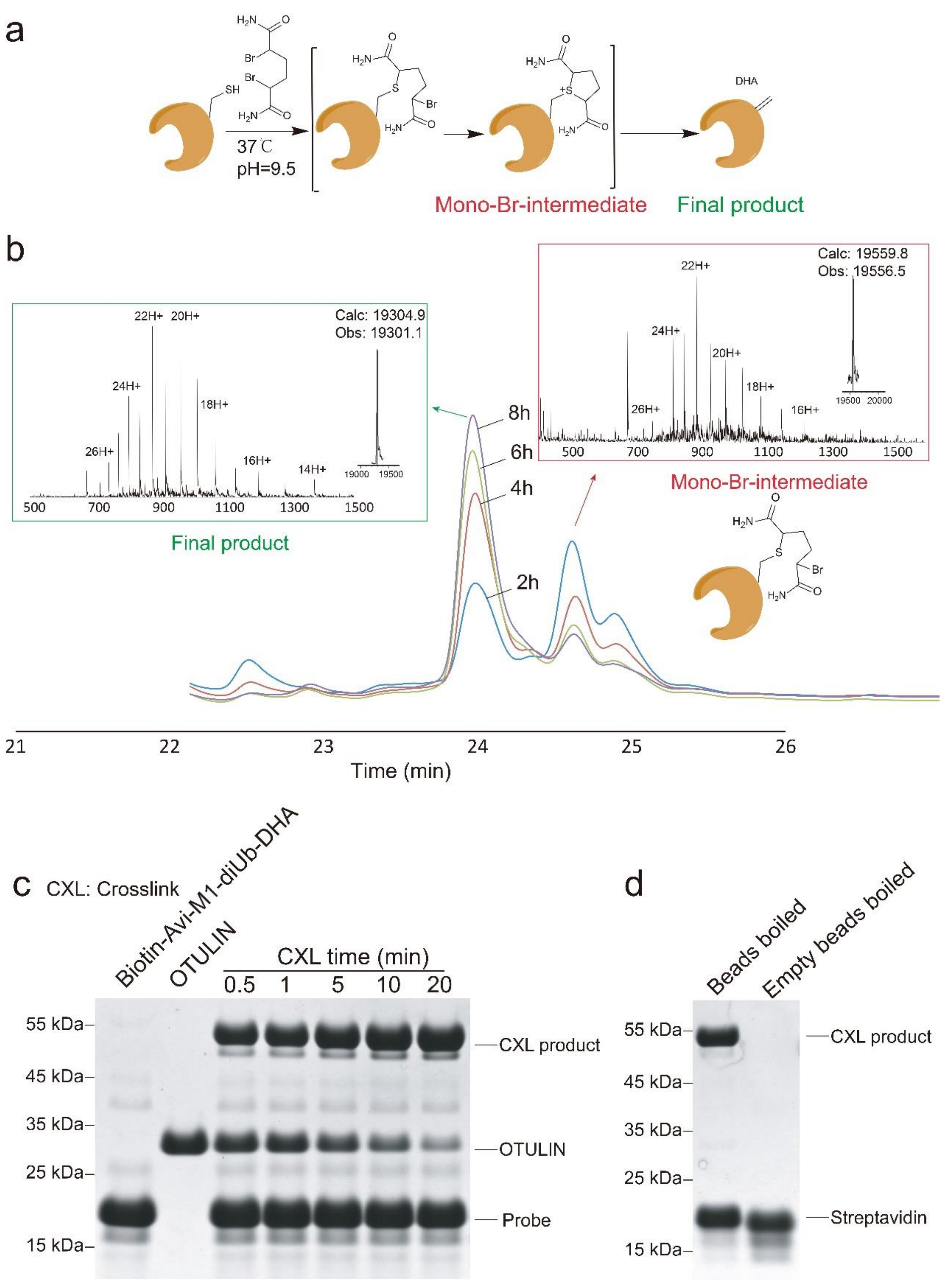

Supplementary Figure 1 One-pot synthesis of Biotin-Avi-M1-diUb-DHA

a, Mechanism of desulfurization; **b**, Time scale tracing of one-pot synthesis of Biotin-Avi-M1-diUb-DHA by HPLC; **c**, Time scale investigation of crosslinking activity of Biotin-Avi-M1-diUb-DHA with OTULIN (N=1 biologically independent experiment); **d**, Binding activity of Biotin-Avi-M1-diUb-DHA to Streptavidin beads (N=1 biologically independent experiment). CXL: crosslink. Source data are provided as a Source Data file.

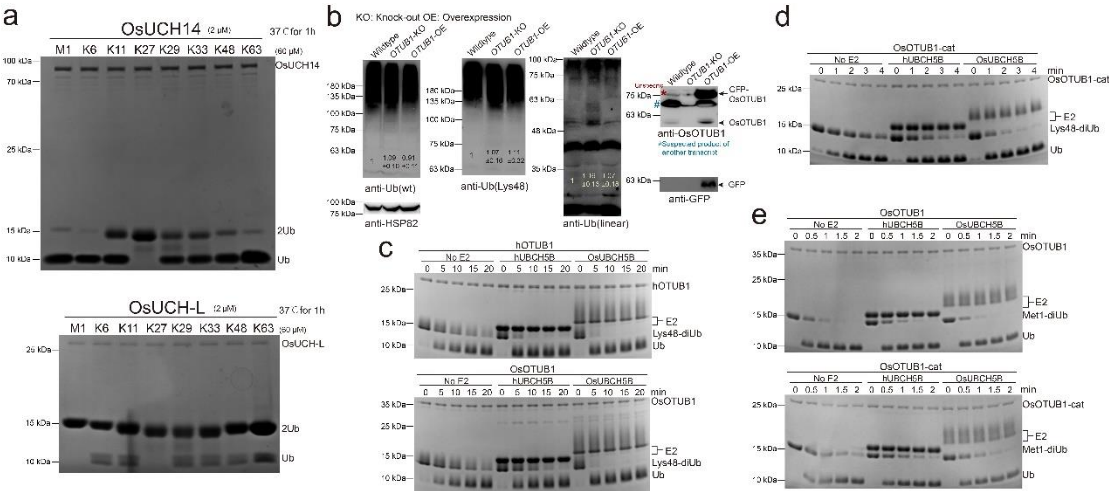

Supplementary Figure 2 Activity test of DUBs *in vitro* or *in vivo*

a, Activity of OsUCH14 and OsUCH-L toward diUbs with 8 linkage types (N=3 biologically independent experiments); **b**, Effects of OsOTUB1 over-expression and knockout on the abundance of mono-Ub, K48- and M1-chain at the young panicle differentiation stage, the numbers indicate the relative amount to the wildtype rice (defined to 1) based on three replicates monitored by ImageJ, * indicates the unspecific band, # indicates the suspected product of another transcript; **c**, Effects of E2 (hUBCH5B/OsUBCH5B) on the activity of hOTUB1 toward K48-diUb (N=3 biologically independent experiments); **d**, Effects of E2 (hUBCH5B/OsUBCH5B) on the activity of OsOTUB1-cat toward K48-diUb (N=3 biologically independent experiments); **e**, Effects of E2 (hUBCH5B/OsUBCH5B) on the activity of OsOTUB1/-cat toward Met1-diUb (N=3 biologically independent experiments). Source data are provided as a Source Data file.

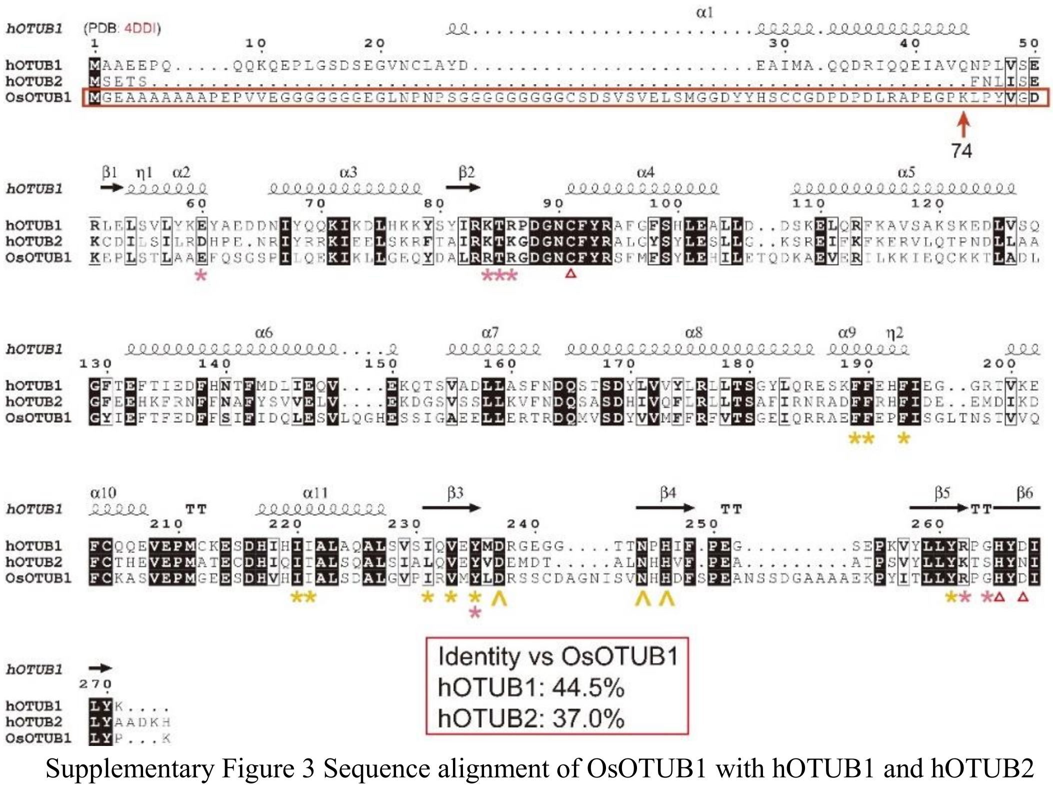

Supplementary Figure 3 Sequence alignment of OsOTUB1 with hOTUB1 and hOTUB2

Sequence included in the red frame indicates the unorganized region; red triangles indicate the catalytic triad in OsOTUB1, hOTUB1 and hOTUB2; red stars indicate the residues in OsOTUB1 responsible for proximal Ub binding; yellow stars and arrowheads indicate the residues in OsOTUB1 responsible for hydrophobically and hydrophilically binding of distal Ub, respectively; red frame contains the sequence identity of OsOTUB1 with hOTUB1 and hOTUB2.

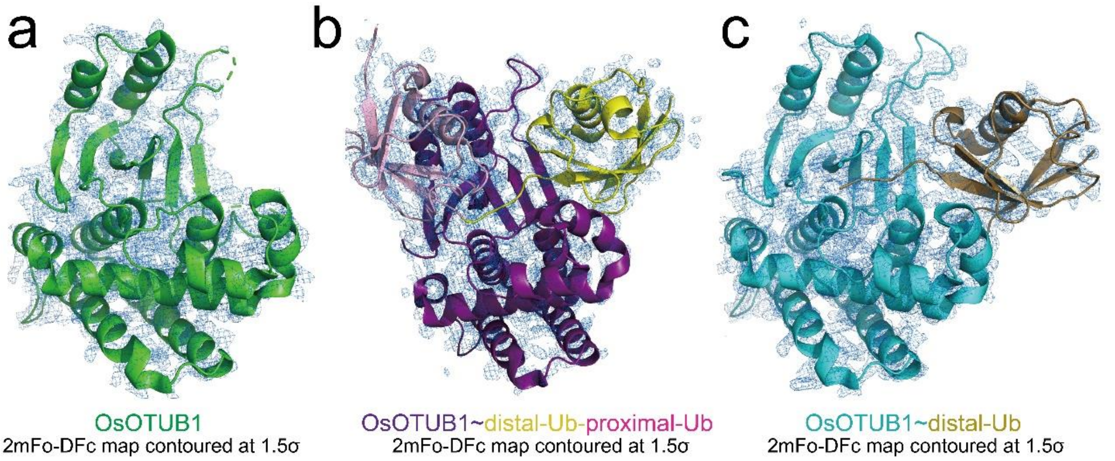

Supplementary Figure 4 Electron density map of structures related to OsOTUB1

a, $2mF_{O}$-DF_{C} map (contoured at 1.5σ) of OsOTUB1; **b**, $2mF_{O}$-DF_{C} map (contoured at 1.5σ) of OsOTUB1 crosslinked with Met1-diUb-DHA; **c**, $2mF_{O}$-DF_{C} map (contoured at 1.5σ) of OsOTUB1 crosslinked with Ub-PA.

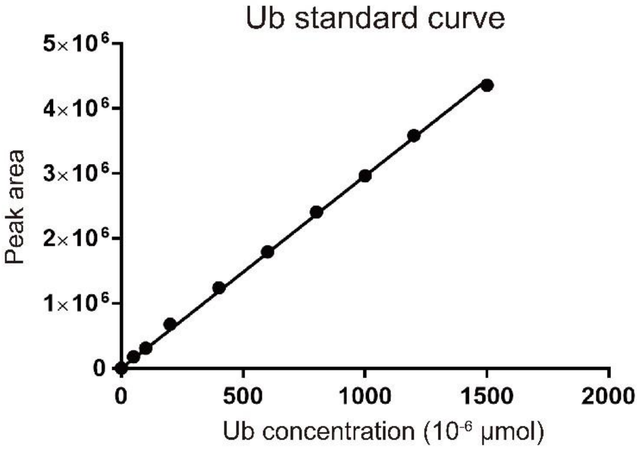

Supplementary Figure 5 Standard curve of Ub

The peak areas of Ub traced by HPLC were plotted against their corresponding molar quantity. Source data are provided as a Source Data file.

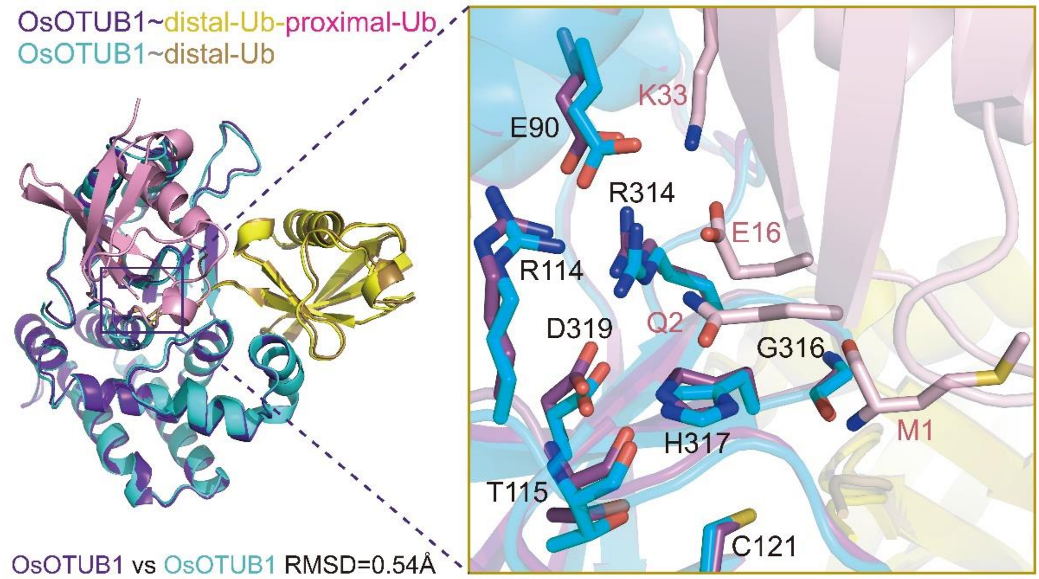

Supplementary Figure 6 Structure alignment of OsOTUB1-cat~Met1-diUb-DHA and OsOTUB1-cat~Ub-PA

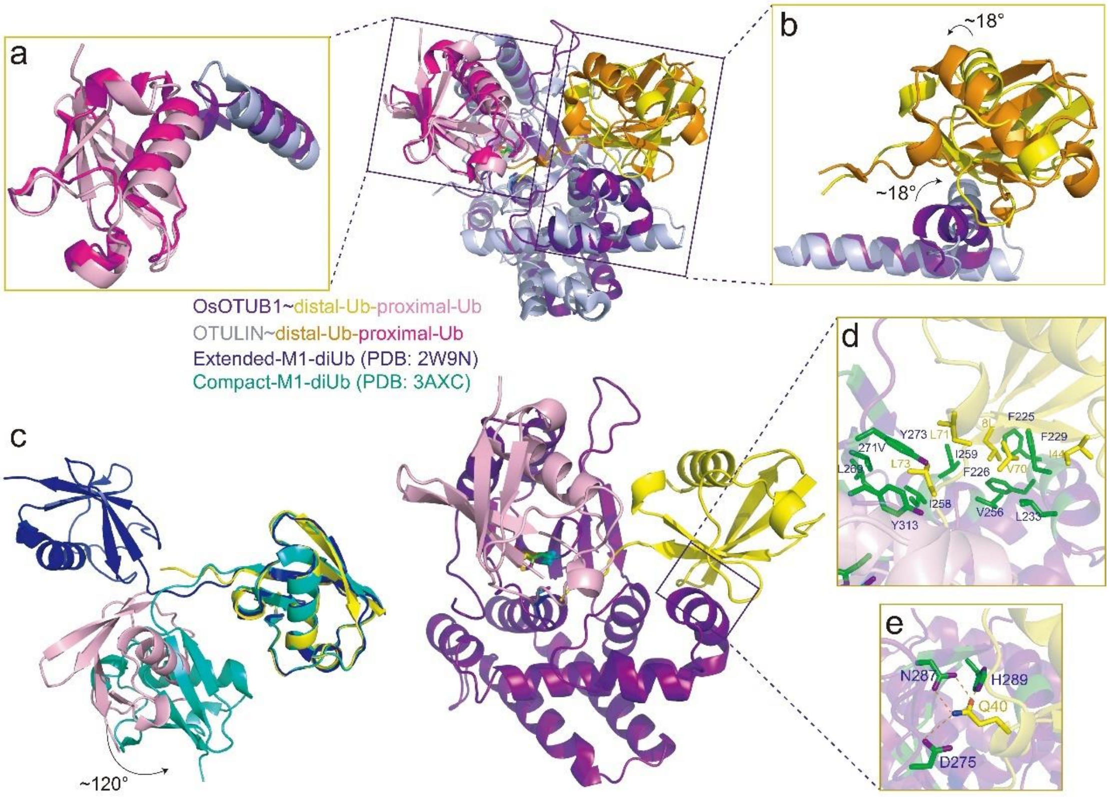

Supplementary Figure 7 Structure alignment

a, Structure alignment between proximal Ubs of OsOTUB1~M1-diUb-DHA and OTULIN(C129A)-M1-diUb; **b**, Structure alignment between distal Ubs of OsOTUB1~M1-diUb-DHA and OTULIN(C129A)-M1-diUb; **c**, Structure alignment of Met1-diUb in OsOTUB1~M1-diUb-DHA with extended and compact Met1-diUbs; **d**, Hydrophobic interaction between S1 pocket and distal Ub; **e**, Hydrogen bond interaction between S1 pocket and distal Ub.

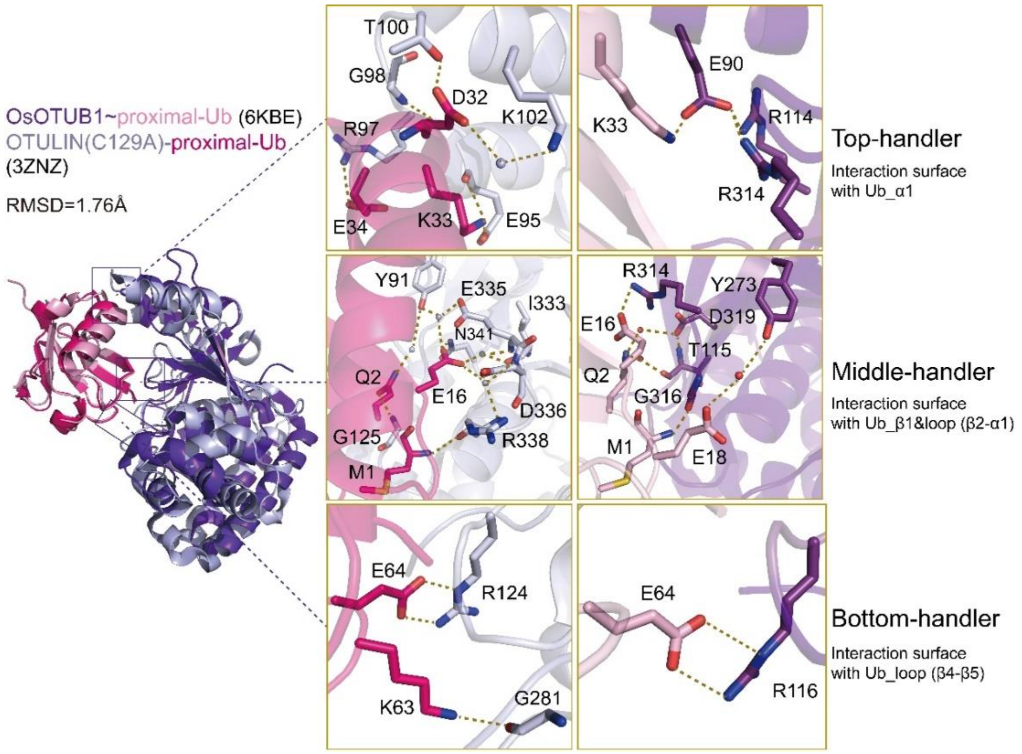

Supplementary Figure 8 Structure comparison of S1' between OsOTUB1~M1-diUb-DHA and OTULIN(C129A)-M1-diUb

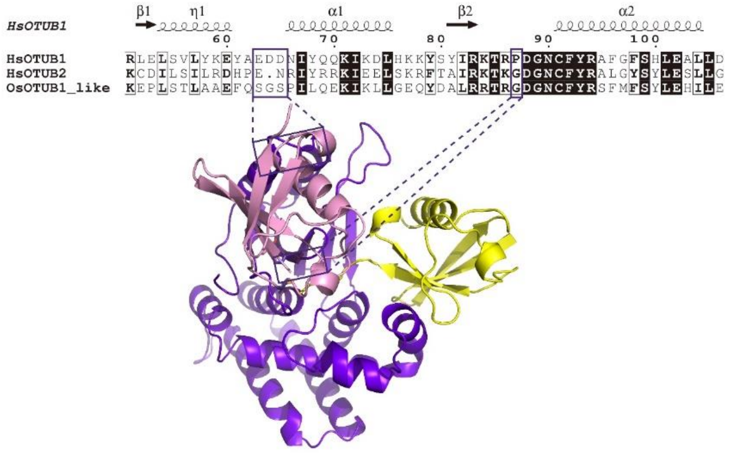

Supplementary Figure 9 Sequence alignment of S1’ from hOTUB1, hOTUB2 and OsOTUB1

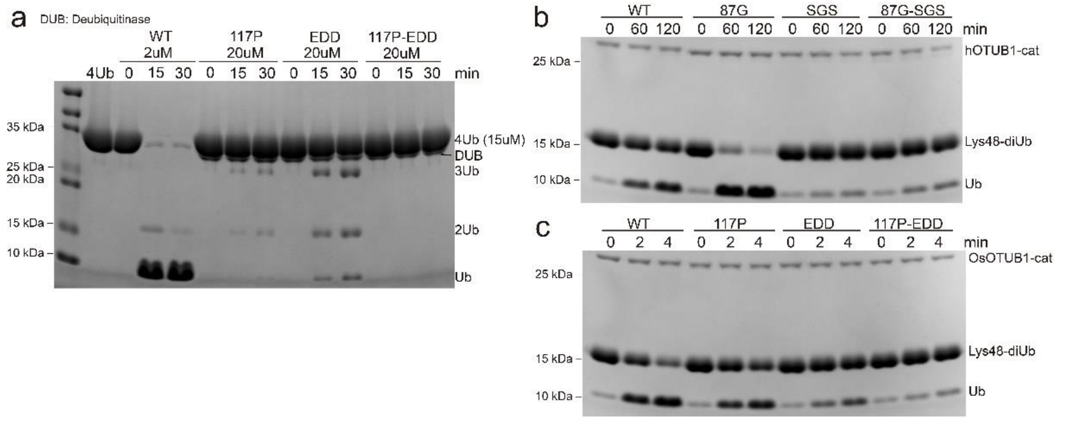

Supplementary Figure 10 Effects of mutations upon N- and C-handle motif on the activity toward M1-tetraUb and K48-diUb

a, Activity of OsOTUB1-cat and its mutants upon N- and C-handle motif against Met1-tetraUb (N=3 biologically independent experiments); **b**, Activity of hOTUB1-cat and its mutants upon N- and C-handle motif against K48-diUb (N=3 biologically independent experiments); **c**, Activity of OsOTUB1-cat and its mutants upon N- and C-handle motif against K48-diUb (N=3 biologically independent experiments). Source data are provided as a Source Data file.

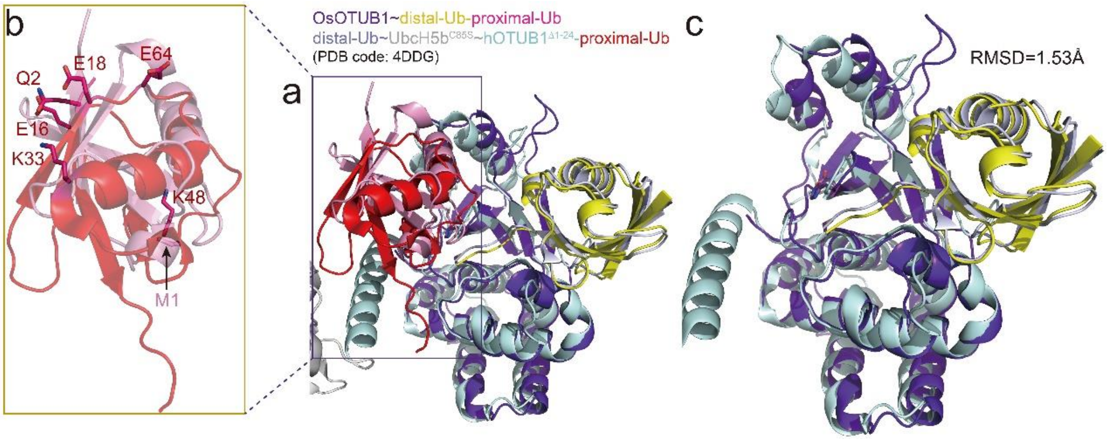

Supplementary Figure 11 Structure alignment between OsOTUB1-cat~M1-diUb-DHA and Ub^{distal}~UbcH5b^{C85S}~hOTUB1$^{\Delta 1\text{-}24}$-Ub^{prox}

a, Structure alignment between OsOTUB1-cat~M1-diUb-DHA and Ub^{distal}~UbcH5b^{C85S}~hOTUB1$^{\Delta 1\text{-}24}$-Ub^{prox}; **b**, Alignment of proximal Ub in OsOTUB1-cat~M1-diUb-DHA with that in Ub^{distal}~UbcH5b^{C85S}~hOTUB1$^{\Delta 1\text{-}24}$-Ub^{prox}, the black arrow indicates Met1; **c**, Alignment of OsOTUB1-cat~distal-Ub with Ub^{distal}~hOTUB1$^{\Delta 1\text{-}24}$.
