# Supplementary material for: Met1-specific motifs conserved in OTUB subfamily of green plants enable rice OTUB1 to hydrolyse Met1 ubiquitin chains
Source: Nat Commun. 2022 Aug 9;13:4672. doi: 10.1038/s41467-022-32364-3 (PMC9363410; doi:10.1038/s41467-022-32364-3)
Supplement: Supplementary file 3 — Description of additional supplementary files [file 41467_2022_32364_MOESM3_ESM.docx]

**Description of Additional Supplementary Files**

File Name: Supplementary Data 1

Description: This supplementary data contains sequences of peptidases in the C65 family (PF10275, update to January 2020), which were analyzed by MEGA-X in the part of “The influence of the N-handle and C-handle motifs on Met1 activity is observed in the OTUB subfamily from other species”.

File Name: Supplementary Data 2

Description: This supplementary data contains alignment for the 149 sequences that incorporated the XGY-G pattern, showing the N-handle, C-handle motif and catalytic triad, which were further used to analyze the evolution pathway of the XGY-G pattern in the part of “The influence of the N-handle and C-handle motifs on Met1 activity is observed in the OTUB subfamily from other species”.

File Name: Supplementary Data 3

Description: This supplementary data is a summarized list of primers, plasmids and cell strains used in this work.
