# Supplementary material for: Met1-specific motifs conserved in OTUB subfamily of green plants enable rice OTUB1 to hydrolyse Met1 ubiquitin chains
Source: Nat Commun. 2022 Aug 9;13:4672. doi: 10.1038/s41467-022-32364-3 (PMC9363410; doi:10.1038/s41467-022-32364-3)
Supplement: Supplementary file 5 — Supplementary Data 2 [file 41467_2022_32364_MOESM5_ESM.pdf]

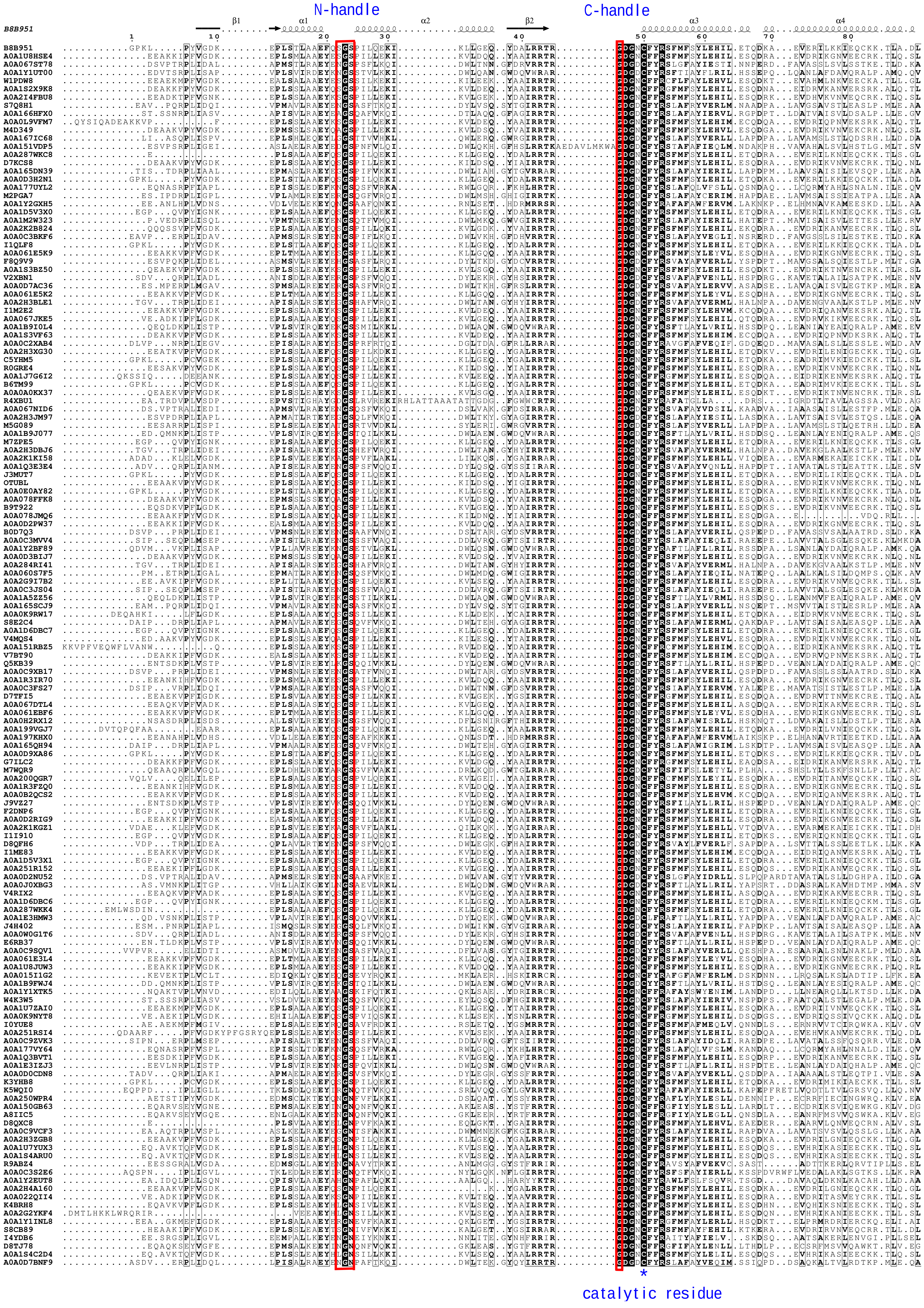
N-handle
C-handle
B8B951
β1
α1
α2
β2
α3
α4
1
10
20
30
40
50
60
70
80
*
catalytic residue

```
                η1      α5                             α6                  α7                        α8                          α9
B8B951
            90        100       110       120       130       140       150       160       170
B8B951      GYIEFTFEDFF......SIFIDQLESVLQGH.ESS.......IGA........EELLERTRDQMVSDY..........VVMFFRFVTSGEIQRRAEFFEPFISGLT...................NSTVVQFCKASV
A0A1U8HSE4  GHTDFTFEDFF......SLFLEQLECVLQGK.EDS.......ISQD........ELILRSRDQSISDY..........VVMFFRFVTSGEIRKRSEFFEPFILGLT...................NATVEQFCKSSV
A0A067ST78  GIEKLVYEDFY......DDFTSLIESITKPG.ADGLVL......N.......GERLLQAFQQAEISNS..........IVIYLRFLTSAQIRLNREAYEGFLVHPDT................KEPMDVDSFCANVV
A0A1Y1UT00  GFDPDLYEEFL......EPLLVLLRGFDADT.PTEW..................TLIQQLQDAERSNC..........IVVALRLVTSAYIRTHEDVFSPFLLSPTT................FLPLTTDDFCRQEV
W1PDW8      GYLEFTFEDFV......LSFTDLLEGVIQGT.EAS.......IS........YDELLDRSRNASISNY..........VVMFFRFVTSAEIKKRADFFEPFIMGLS...................NVTVEKFCQSSV
A0A1S2X9K8  GYAELTFEDFF......TLFLEQLEDVIQGK.ETS.......ISHE........ELVLRSRDQSVSDY..........VVMFFRFVTSAEIQKRSEFFEPFIMGLT...................NTTVEQFCKSAV
A0A2I4FBU8  GYADFTFEDFF......ALFLEQLESVLQGN.ETS.......ISQD........ELLLRSRDQSISDY..........VVMFFRFVTSGEIRRRSEFFEPFIMGLT...................NTTVEQFCKSSV
S7Q8H1      GFQKLVFEDFY......DTFVSIIKRIVQPE.ADGTTL......T........PLLLEAFQNPEVSNS..........VVVYLRLLTSAQIRTDPDSYQDFLFHPEL................GELMTPREFCETFV
A0A166HFX0  GFDKFAYEDFY......EELLGLLKRIGPDA.RNPL.T......P........SALLQAFQAPEVSTA..........IVVFLRLLTSAQIRMDPDSFDAFLFHPDF................GNQMTARDFCETFV
A0A0L9VFM7  GYADLTFEDFFAYSQCLKYMFTVFKKMVGSGGLDP.......ISSH........ELVIRSRDQSISDY..........VVMFFRFVTSGEIQMRTEFFEPFILGLT...................NTTVEQFCKLSV
M4D349      GYTDFTFEDFF......ALFLEQLDDILQGG.EES.......IRSRDQSVSDYSKLLNFFDNSYSKECGFDK.FLCLSVVMFFRFVTAGEIKTRAEFFEPFITGLS...................NTTVDQFCKTSV
A0A167IC68  GFQELVYEDFY......DVLASLINGIVSPL.SPTSSL......LS......GSQLLAAFQDAETSNS..........IVVYLRLLTSAYIRAHEEDFLPYLFSPDT................GDLLDVREFCEREV
A0A151VDP5  GFQSLVFDDFY......DVFVAIIRNIVKPD.QDKKIL......T.......PARLLDAFQSPETSNS..........IVVYLRLVTSAQMRLAADEYDGLLFHPET................GEPMEVRTFCENFV
A0A287WKC8  GYIEFTFEDFF......SMFIEELQNVLQGH.GTS.......IGP........EELLERTRDQTTSDY..........VVMFFRFVTSGEIQRRAEFFEPFISGLT...................NSTVVQFCKSSV
D7KCS8      GYTDFTFEDFF......ALFLEQLDDILQGT.EES.......IS........YDELVNRSRDQSVSDY..........IVMFFRFVTAGDIRTRADFFEPFITGLS...................NTTVDQFCKSSV
A0A165DN39  GFQKLVYEDFY......DTLVSLINSIVTPD.SNGKVL......T.......QTTLLEAFNTPEVSNS..........IVVYLRLLTSAQMRSEPEKYADVLFHPEL................DEPMELREFCENFV
A0A0D3H2N1  GYIEFTFEDFF......SIFIDQLESVLRGH.ESS.......IGA........EELLERTRDQMVSDY..........G....LFVTSGEIQRRAEFFEPFISGLT...................NSTVVQFCKASV
A0A177UYL2  GYDRSVTEDFW......EPLRDLLSRLPGCT.PAVQAP......PL.....DTDSLVLAFNDAETTNS..........IVVFLRLSLSAWLKLNADKFSVFLFALDSAS............GSGEPPTMDEFCSQEV
M2PGA7      GFQPLVFEDFY......EMFLSLVRKILAPD.ENGKVL......T.......PAALLGAFNDPETSNS..........IVVYLRMLTSAQIRSDPDI.SNFLLSPET................AEFVEPRVFCEHFV
A0A1Y2GXH5  QFELLAFEDFY......LVTLETLQNLVHYT.P...........E.........ELLAAFQNDEISNS..........IVMHFRLMVSAYLKTHQEDYAPFLEFG.....................QTMDEFCSMHV
A0A1D5V3X0  GYIEFTFEDFF......SGHETSIGP...........................EELLERTRDQTTSDY..........VVMFFRFVTSGEIQRRAEFFEPFISGLT...................NSTVVQFCKSSV
A0A1M2W323  GFQKLVFEDFY......DDFLGLINAVIQPR.QDGQLL......T.......INSLLEAFNVPEVSNS..........VVMYLRMLTSAEIKSEAMEYSPFLFNPET................TEPMDPESFCNNFV
A0A2K2B824  GYVDFTFEDFF......ALFLEQLDDVLQGN.ETS.......ISHE........ELLNRSRDQSVSDY..........VVMFFRFVTSGEIRRRSEFFEPFVFGLT...................NTTVEQFCKSSV
A0A0C3BKF6  GIEKMVYEDFY......EDFETLIQSIIKPG.KDGRTL......N.......TQQLLAFQQPEVSNS..........IVIYLRFLTSAQIRLNRDSYEGFLVHPDT................KDPMDVDSFCANVV
I1QLF8      GYIEFTFEDFF......SIFIDQLESVLQGH.ESS.......IGA........EELLERTRDQMVSDY..........VVMFFRFVTSGEIQRRAEFFEPFISGLT...................NSTVVQFCKASV
A0A061E5K9  GYADFTFEDFF......ALFLEQLDSVLQGN.EDS.......ISQD........ELIIRSRDQSISDY..........VVMFFRFVTSGEIRKRSEFFEPFILGLT...................NATVEQFCKSSV
F8Q9V9      RFDPFVYEDFM......ECFTTLIQSIVVPD.REGLTL......T.......PQSLLDAFQQPESSNS..........IVVYLRLLTSAQIRADPDAYAGFLHDPDN................YMELQLETFCQHYV
A0A1S3BZ50  GYTEFTFEDFF......ALFLEQLESALQGN.ESS.......ISHD........ELVIRSRDQSISDY..........VVMFFRFVTSGEIQKRSEFFEPFIMGLT...................NGTVDQFCKTAV
V2XBN1      GFEKLVFEDFY......EVLETLAGNIVTPG.PAGTTL......D.......DTTLLAVFQNNTTSNS..........IVMYLRMLTSAQLRLDPDSYAPFLFHPEL................GEPMEVREFCEHFV
A0A0D7AC36  GFEDMVFEDFY......DELLRLVNQVVIPN.EEGALL......T.......MEGLLEAFCAPEISNS..........IVFYLRLITSAKIRRDSDVFAPFLMHPET................GEMMAPREFCEHFV
A0A061E5K2  GYADFTFEDFF......ALFLEQLDSVLQGN.EDS.......ISQD........ELIIRSRDQSISDY..........VVMFFRFVTSGEIRKRSEFFEPFILGLT...................NATVEQFCKSSV
A0A2H3BLE1  GFQKLVFEDFY......DVLESLLLSIVTPQ.TTGKTL......N.......AVGLLEAFQSPEVSNS..........IVVYLRLLTSAQIRIDPDAFAPFLFHPEL................GEPMEVREFCEHFV
I1M2E2      GYADLTFEDFF......ALFLEQLESVIQGK.ETS.......ISHE........ELVLRSRDQSVSDY..........VVMFFRFVTSAAIQKRTEFFEPFILGLT...................NTTVEQFCKSSV
A0A067JKE5  GYADFTFEDFF......ALFLEQLDCVLQGN.ETS.......VNHD........ELINRSRDQSISDY..........VVMFFRFVTSGEIRRRSDFFEPFILGLT...................NATVEQFCKSSV
A0A1B9I0L4  NFQKDLYEEFL......DPLLVLIRSFAEGG.DDSTST......E........YGIVQALQDPERSNC..........IVVALRLITSSYIRTHSELFSPFLLSPTT................FLPLSTDEFCKSEV
A0A1S3VF63  GYADLTFEDFF......ALFLEQLECVIQGK.EMS.......ISHE........ELVIRSRDQSISDY..........VVMFFRFVTSAEIQMRTEFFEPFILGLT...................NTTVEQFCKLSV
A0A0C2XAB4  GFQRLVYEDFY......DTLCGLIQNIATPE.MNGSVL......T.......EATLLDQFQHPEASNS..........IVAFLRLLTSSHLRLNEEEYSPFLFHPEL................GEPMNVRDFCENFV
A0A2H3XG30  GYVDFTFEDFF......SIFMEQLESVLQGN.QTS.......ISHE........ELLLRSRDQMISDY..........VVMFFRFVTSGEIRKRAEFFEPFIAGLT...................NSTVDQFCKASV
C5YHM5      GYIEFTFEDFF......AIFIDMLESVLQGH.ETP.......IGP........EELLERTRDPQVSDC..........VVMFFRFVTSGEIQRRSDFFEPFISGLT...................NSTVVQFCKASV
R0GRE4      GYTDFTFEDFF......ALFLEQLDDILQGN.EGS.......IS........YDELVNRSRDQSVSDY..........IVMFFRFVTAGDIRTRADFFEPFITGLS...................NTSVDQFCKSSV
A0A1J7G6I2  GYPDLTFEDFF......SLFLEELDCVIQGK.ENS.......ISHE........ELVLRSRDQSISDY..........VVMFFRFVTSAAIQKRSEFFEPFILGLS...................NTTVEQFCKSSV
B6TM99      GYIEFTFEDFF......SIFIELLESVLQGH.ETP.......IGP........EELLERTRDPQVSDY..........VVMFFRFVTSGEIQRRSDFFEPFISGLT...................NSTVVQFCKASV
A0A0A0KX37  GYTEFTFEDFF......ALFLEQLESALQGN.ESS.......ISHE........ELVIRSRDQSISDY..........VVMFFRFVTSGEIQKRSEFFEPFIMGLT...................NGTVDQFCKTAV
R4XBU1      GFSPLVYEDFL......DGFTRLVRRAR......T.......V..........ESVLAAMNEPEESNS..........AVVLLRFLTSAYVKAHPEGYAGYLLDG.....................EVLTDWCERWI
A0A067NID6  GFQKLVFEDFY......DVLASLLQNIAQGT.NDGKSL......D........SKILLAFQSPEISNS..........IVVYLRLLTSARIRTDPNEYAPFLFHPEI................GIQLEPREFCEAFV
A0A2H3JM97  GFDRMAFEDFY......DCFVNLIKRVITPD.NAGQLL......S.......SSMLLEEFNMPEVSNS..........VVVYLRLLTSAHIRADPDSYIPFLLDPDT................GELNDLQQFCISQV
M5G089      GFQQLVYEDFY......DVLVRLISSIISPP.GPYEPL......LS......SPGLLAAFQDAETSNS..........IVVYLRLLTSGYIRAHEEEFLPYLFSPDT................GDLLDTRDFCEREV
A0A1B9J077  GFDKVLYEEFL......DPLLALIRSFAEEG.ESTSTE......Y.........GIVQSLQDPERSNC..........IVVALRLITSSYIRTHSDLFSPFLLSPTT................FLPLSTDDFCRSEV
M7ZPE5      GYIEFTFEDFF......SMFIEELQNVLQGH.ETS.......IGP........EELLERTRDQTTSDY..........VVMFFRFVTSGEIQRRAEFFEPFISGLT...................NSTVVQFCKSSV
A0A2H3DBJ6  GFQKLVFEDFY......DVLESLLLSIVKPQ.TTGRTL......N.......AVGLLEAFQSPEVSNS..........IVVYLRLLTSAQIRIDPDAFSPFLFHPEL................GEPMEVREFCEHFV
A0A2K1KI58  GQAEFTFEDYL......AIFVEQLHGVIQGK.ETS.......TSL........ETLVERCRDQYVSNC..........VVMFFRFVTSGEIRRRSEFFEPFIQGMS...................NMSVVQFCRSCV
A0A1Q3E3E4  GFEKMVFEDFY......EVLEELVKSVVTPN.ASGSIL......T.......DEGLLETFQDPEKSNS..........IVVYLRMLTSAQIRLDPENFAPFLFHPEL................GEPMEIRDFCEHFV
J3MUT7      GYIEFTFEDFF......SIFIDQLESVLQGH.ETS.......IGA........EELLERTRDQMVSDY..........VVMFFRFVTSGEIQKRAEFFEPFISGLT...................NSTVVQFCKASV
OTUBL       GYTDFTFEDFF......ALFLEQLDDILQGT.EES.......IS........YDELVNRSRDQSVSDY..........IVMFFRFVTAGDIRTRADFFEPFITGLS...................NATVDQFCKSSV
A0A0E0AY82  GYIEFTFEDFF......SIFIDQLESVLQGH.ESS.......IGA........EELLERTRDQMVSDY..........G....LFVTSGEIQRRAEFFEPFISGLT...................NSTVVQFCKASV
A0A078FFK8  GYTDFTFEDFF......ALFLEQLDDILQGG.EES.......IS........YDELVNRSRDQSVSDY..........IVMFFRFVTAGEIKTRAEFFEPFITGLS...................NTTVDQFCKTSV
B9T922      GYVDFTFEDFF......ALFLEQLDSVLQGN.ETS.......ISHD........ELINRSRDQSISDY..........VVMFFRFVTSGEIRKRSEFFEPFILGLT...................NATVEQFCKSAV
A0A078JMQ6  ...DFTFEDFF......ALFLEQLDDILLGS.DES.......IS.Y.......EELVYRSRDQSVSDY..........IVMFFRFVTAGEIRTRAEFFEPFITGLS...................NSTVDQFCKISV
A0A0D2PW37  GHTDFTFEDFF......SLFLEQLECVLQGN.EDS.......ISQD........ELILRSRDQSISDY..........VVMFFRFVTSGEIRKRSEFFEPFILGLT...................NATVEQFCKSSV
B0D7Q3      GIDKIVYEDIY......DEFVGLISTIVQPN.SAGKKL......D.......SAGLLAFQSPEMSNA..........VVVYMRFLTSAEIRVHRENYEAFLIHPET................HEPMGVDAFCANIV
A0A0C3MVV4  SFQEIVYDLPY......GVFTDLIKRIISQS.GDLL.T......S.......AGLLETFQDSEAPQY..........IVMYLRLLTSAQIRTAPEEFEEFIMHPDT................GMKMTVTEFCENFV
A0A1Y2BF89  NFDSDLYEEFL......DPLLAIVRSLAEDT.APPTE................YALIQSLQDAEKSNC..........IVVALRLITSAYIRANPTTFEPFLLSPTT................FLPLTVDDFCRQEV
A0A0D3BIJ7  GYTDFTFEDFF......ALFLEQLDDILQGG.EES.......IS........YDELVNRSRDQSVSDY..........IVMFFRFVTAGEIKTRAEFFEPFITGLS...................NTTVDQFCKTSV
A0A284RI41  GFQKLVFEDFY......DVLESLLLSIVTPQ.TTGKTL......N.......AVGLLEAFQSPEVSNS..........IVVYLRLLTSAQIRIDPDAFAPFLFHPEL................GEPMEVREFCEHFV
A0A060S7F5  GFQELVFEDFH......DAFADLIKAVVEPR.VDGRLL......T.......ISSLLKAFNEPETSNS..........VVMYLRLLASAHIKGNPDSYAGFLLHPET................AESMDPESFCNNFV
A0A2G9I7B2  GYAEFTFEDFF......ALFLEQLESVLSGN.EAS.......ISHE........ELVQRSRDQSISDC..........VVMFFRFITSGELKKRSEFYEPFVQGLT...................NGTVEQFCKSSV
A0A0C3JS04  GFQEIVYDLPY......GVFTDLIKRIISQS.GDLL.T......S.......AGLLETFQDSEAPQY..........IVMYLRLLTSAQIRTAPEEFEEFIMHPDT................GMKMTVTEFCENFV
A0A1A5ZZ56  NFQKDLYEEFL......DPLLALVRSFAEGG.EDSTST......E........YGIVQALQDPERSNC..........IVVALRLITSAYIRTHSELFSPFLLSPTT................LLPLSTDEFCKAEV
A0A165SCJ9  GFQPLVYDDFY......ETLSSIIRRVVQPD.DSGTTL......T.......LPLLLEAFQSPEVSNS..........VVVFLRLLTSAQIRTDPDEFAPFLFHPEI................GEPMTPREFCETFV
A0A0K9RW17  GYADFTFEDFF......ALFLEQLESVVQGA.DAS.......ISHD........ELVERSRDQSISDY..........VVMFFRFVTSGEIRKRSEFFEPFILGLT...................NATVDQFCKSSV
S8E2C4      GFQPLVFEDFY......DCLVALIKQIIAPS.GDGRLL......T.......PATLLEAFNSPETSNS..........IVVYLRLLTSAQIRADPDAYAGFLMHPEI................GEPMELRDFCESFV
A0A1D6DBC7  GYIEFTFEDFF......SMFIEELQNVLQGH.ETS.......IGP........EELLERTRDQTTSDY..........VVMFFRFVTSGEIQRRAEFFEPFISGLT...................NSTVAQFCKSSV
V4MQS4      GYTDFTFEDFF......ALFLEQLDDILQGT.EES.......IS........YEELVNRSRDQSVSDY..........IVMFFRFVTAGEIRTRAEFFEPFITGLS...................NTTVDKFCKSSV
A0A151RBZ5  GYADLTFEDFF......SVTSEQVEPLEASC.............H........EELVLRSRDQPTSDY..........VVMFFRFVTSAEIQKRKEFFEPFILGLT...................NTTVEQFCQSSV
V7BT90      GYADLTFEDFF......ALFLEQLECVIQGK.ETS.......ISHE........ELVIRSRDQSISDY..........VVMFFRFVTSAEIQMRTEFFEPFILGLT...................NSTVEQFCKLSV
Q5KB39      GFQKDIYEEFL......DPLLALIRSFAESD.ETTANE......Y........SIVQALQDPERSNY..........IVVSLRLITSSYIRTHADLFSPFLFSPTT................FLPLSTEEFCRQEV
A0A0C9XB17  GIDKIVYEDIY......DEFVGLIGTIVQPN.SAGKKL......D.......SAGLLAFQSPEMSNA..........VVVYMRFLTSAEIRVHRENYEAFLIHPET................HERMGVRAFCANIV
A0A1R3IR70  GYADFTFEDFF......ALFLEQLDSVLQGN.EDS.......ISQD........ELIIKSRDQSISDY..........VVMFFRFVTSGEIRKRSDFFEPFILGLT...................NATVEQFCKSSV
A0A0C3FS27  GFQKMVFEDFY......DILVSLVHQVVQPD.HEGKIL......S.......PELLLEAFQHPEGKFSMMRSLLRLSTSVVVYLRLLTSAQIRADPDEYAPFLFHPEL................GIQMEPQEFCENFV
D7TFI5      GYADFTFEDFF......ALFLEQLESVVQGK.ETS.......ISHD........ELVIRSRDQSISDY..........IVMFFRFVTSGEIKRRSEFFEPFILGMTN.................ATATVEQFCKSSV
A0A067DTL4  GHADFTFEDFF......ALFLEQLDNVLQGN.EFS.......ISHG........ELVVRSRDQSVSDY..........VVMFFRFVTSGEIRKRSEFFEPFILGLT...................NATVEQFCKSSV
A0A061EBF6  GYADFTFEDFF......ALFLEQLDSVLQGN.EDS.......ISQD........ELIIRSRDQSISDY..........VVMFFRFVTSGEIRKRSEFFEPFILGLT...................NATVEQFCKSSV
A0A0H2RX12  GFEKMVYEDFH......DVIVSIVRQIVVPE.PSGATL......S.......PALLLEAFQDAEVSNC..........IVVFLRFLTSATIKTDPDSYLPYLFDPET................GDDISLTDFCNRHV
A0A199VGJ7  GYADFTFEDFF......AIFIEQLESILQGN.ETS.......ISEE........ELLQRSCDQMISDY..........VVMFFRFVTSGEIRRRAEFFEPFIAGLT...................HSTVDQFCKASV
A0A197KHX0  QFELLAFEDFY......QVTLETLQNLV..H.FTS.........E.........ELLTAFQNDEISNS..........IVMHFRLVASAFLKTHQDDYAPFLEFG.....................QTMDEFCSMHV
A0A165QH94  GFQRMVFEDFY......DCLVSLINEIITPE.RDGRLL......T.......PAILLEAFNSPEVSNS..........IVVYLRLLTSAKIRADPDEYAAFLFHPEI................GEPMEVRDFCESFV
A0A0D9XA86  GYIEFTFEDFF......SIFIDQLESVLQGH.ETS.......IGA........EELLERTRDQMVSDY..........VVMFFRFVTSGEIQRRAEFFEPFISGLT...................NSTVVQFCKASV
G7ILC2      GYAELTFEDFF......TLFLEQLEDVIQGK.ETS.......ISHE........ELVLRSRDQSVSDY..........VVMFFRFVTSAEIQKRSEFFEPFIMGLT...................NTTVEQFCKSAV
M7WQR9      GFEQMVWEDFW......EPLRDLLYQMGPEE.KD...GGKRKDGMERL...TRDGLMKAFNDAETNSC..........IIVILRLITSAYLRTHSDEFSPFLFALENDPR..........FAADGGITMKDFCEREV
A0A200QGR7  GYADFTFEDFF......ALFLEQLESVLQGN.ETS.......ISHD........ELVRRSRDQSVSDY..........VVMFFRFVTSGEIRRRSEFFEPFILGLS...................STTVEQFCKTSV
A0A1R3FZQ0  GYADFTFEDFF......ALFLEQLDSVLQGN.EDS.......ISQD........ELIIKSRDQSISDY..........VVMFFRFVTSGEIRKRSDFFEPFILGLT...................NATVEQFCKSSV
A0A0B2QCS2  GYADLTFEDFF......ALFLEQLESVIQGK.ETS.......ISHE........ELVLRSRDQSVSDY..........VVMFFRFVTSAAIQKRTEFFEPFILGLT...................NTTVEQFCKSSV
J9VZ27      GFQKDIYEEFL......DPLLALIRSFAESG.ETTANE......Y........SIVQALQDPERSNY..........IVVSLRLITSSYIRTHTDLFSPFLFSPTT................FLPLSTEEFCRQEV
F2DNP6      GYIEFTFEDFF......SMFIEELQNVLQGH.GTS.......IGP........EELLERTRDQTTSDY..........VVMFFRFVTSGEIQRRAEFFEPFISGLT...................NSTVVQFCKSSV
A0A0D2RIG9  GHTDFTFEDFF......S.LECVLQGNEDSI.SQD...................ELILRSRDQSISDY..........VVMFFRFVTSGEIRKRSEFFEPFILGLT...................NATVEQFCKSSV
A0A2K1KGZ1  GFAEFTFEDFL......AIFVEQLQSVIQDK.EVS.......VSL........ETLVERCRDQYISNS..........VVMFFRFVTSGEIGRRTEFFEPFIQGMS...................NMSVVQFRRSSV
I1I910      GYIEFTFEDFF......SIFIEQLENVLQGH.ETS.......IGP........EDLLERTRDQMVSDY..........AVMFFRFVTSGEIQRRTEFFEPFISGLT...................NSTVAQFCKASV
D8QFH6      GFEDIVVSGFY......EPLEELIRNIVQPV.DGEMLT......L.......TGLLKAFQEPMTSNS..........IVTYLRMLTSAKIRTEPDDFAPFLIHPEL................GEMMDPREFCETNV
I1ME83      GYADLTFEDFF......ALFLEQLESVIQGK.ETS.......ISHE........ELVLRSRDQSISDY..........VVMFFRFVTSAEIQKRAEFFEPFILGLT...................NTTVEQFCKSSV
A0A1D5V3X1  GYIEFTFEDFF......SMFIEELQNVLQGH.ETS.......IGP........EELLERTRDQTTSDY..........VVMFFRFVTSGEIQRRAEFFEPFISGLT...................NSTVVQFCKSSV
A0A251R152  GYADFTFEDFF......ALFLEQLDSVLQGN.EAS.......ISHD........ELILRSRDQSVSDY..........VVMFFRFVTSGEIRKRAEFFEPFILGLT...................NATVEQFCKSSV
A0A0D2NU52  GIERLVYEDYY......DLLKELVEKAGKGM.RQE...................ELLETFQTPENSNA..........VVLYLRLLTSAQIRLNRDNYEGFLVHPDT................KELMSVDSFTANVV
A0A0J0XBG3  GFQQDLVDEFV......EPLISVISTFVSQP.AGSEPT......E.......FSILEVLQDAEKSNS..........IVVALRLMTAAYIRTHPDDFAPFLFSPAT................LEPVTPEQFCREEV
V4RIX2      GHADFTFEDFF......ALFLEQLDNVLQGN.ESS.......ISHG........ELVVRSRDQSVSDY..........VVMFFRFVTSGEIRKRSEFFEPFILGLT...................NATVEQFCKSSV
A0A1D6DBC6  GYIEFTFEDFF......SMFIEELQNVLQGH.ETS.......IGP........EELLERTRDQTTSDY..........VVMFFRFVTSGEIQRRAEFFEPFISGLT...................NSTVTQFCKSSV
A0A287WKK4  GYIEFTFEDFF......SMFIEELQNVLQGH.GTS.......IGP........EELLERTRDQTTSDY..........VVMFFRFVTSGEIQRRAEFFEPFISGLT...................NSTVVQFCKSSV
A0A1E3HMW3  GFQRDIYEEFL......DPLITILRAFSEGL.ESTANE......Y........SIIQTLQSPELSNS..........IVVSLRLITSAYIRTHADLFSPFLFSPVT................FEPLSAEEFCKQEV
J4H402      GFQRLVFEDFY......DCLVSVINKVLTPE.GDGQLL......S.......PVTLLEAFNSPEVSNS..........IVVYFRLLTSAQIRADSEQYASFLFHPEI................GEPMDLRDFCENFV
A0A0W0G1T6  GFEKLVFEDFY......EVLETLAGNIVTPG.PAGTTL......D.......DTTLLAVFQNNTTSNS..........IVMYLRMLTSAQLRLDPDSYAPFLFHPEL................GEPMEVREFCEHFV
E6RB37      GFQKDIYEEFL......DPLLALIRSFAESG.ETTANE......Y........SVVQALQDPETSNY..........IVVSLRLITSSYIRTHAELFSPFLFSPTT................FLPLSTEEFCKQEV
A0A0C9SQV1  GFQKMVYEDFY......EYLTSIIQQIITPD.PSTGQK......LT......TRILLERFQSAEVSNS..........VVVFLRLLTSAQIRTDPDSYAPFLFHPEL................GIQMEPREFCESFV
A0A061E3L4  GYADFTFEDFF......ALFLEQLDSVLQGN.EDS.......ISQD........ELIIRSRDQSISDY..........VVMFFRFVTSGEIRKRSEFFEPFILGLT...................NATVEQFCKSSV
A0A1U8JUW3  AHTDFTFEDFF......SLFLEQLECVLQGN.EDS.......ISQD........ELILRSRDQSISDY..........VVMFFRFVTSGEIRKRSEFFEPFILGLT...................NATVEQFCKSSV
A0A015I1G2  GYQELVYQDIY......EEHIEKQMKIVANG.EYDE........D.........MLLTIFQTDEISNC..........IVFYLRLITAAYLKLHREEYEPFLGFE.....................FGMDQFCSSSV
A0A1B9FWJ4  GFDKELYEEFL......DSLLVLIRSFAEGG.ESTSTE......Y.........GIVQSLQDAERSNC..........IVVALRLITSSYIRTHSDLFSPFLLSPTT................FLPLSTDDFCRSEV
A0A1Y1XTK5  NFEPLAYEDFY......DVALETLDSLPQQN.SS.....................TLLSIFQTDHISNA..........IVVHFRFITSAYLKLHAEEYLPFIDYA.....................GSMDQFCSHYV
W4K3W5      GFQKIVVEDFY......EVMEGLIKRVVTPD.SRGHYL......T.......PNLLLEAFQDPETSNY..........VVVFLRLLTSAQIRTDPESFEPFLFHPDL................GEPLSIRDFCEGFV
A0A1U7ZAI0  GYADFTFEDFF......ALFLEQLESVLQGS.DSS.......ISHD........ELVTRSRDQSISDY..........VVMFFRFVTSGEIRRRSDFFEPFILGLT...................NASVEQFCKSSV
A0A0K9NYT8  GYAEFTFEDFF......MLFIEQLQCVFNGH.GDS.......YGHD........ELLKRSQDPSVSNY..........VVMFLRFVTTGEIRRRSDFYEPFILGIS...................DSSVDQFCKTAV
I0YUE8      GFQEMVFEDAM......EIVIDQLNSLGTAE.PLS.......V..........PQLEDNFCDAMLSPM..........IIMLLRMLTSAEIQRRSDFFAPFIMGMCD.................DEVSVEQFCRRYV
A0A251RSI4  GYADFTFEDFF......SLFIEQLDSVLQGN.ELS.......ISHD........ELVSRSRDQCVSDY..........VVMFFRFVTSGEIRKRSEFFEPFILGLS...................NTTVEQFCKTSV
A0A0C9ZVK3  NFDEIVYDLPC......GVFTDLIKRIVSQS.SDRL.T......P.......TSLLETFQDPDAPQY..........IVMYLRLLTSAQIRTAPDEFEAFIMHPET................GMEMSITEFCELFV
A0A177VY64  MYDRSVTEDFW......APLRDLLSRIPGCT.PTVQGP......PL.....DTDSLLTAFNDAETTNC..........IVVFLRLCLSAWLKLNTEKFGVFLFALDSAS............GTGEPPTMDEFCSQEV
A0A1Q3BVT1  GYADFTFEDFF......GLFLEQLESVLQAN.DTS.......ISYD........ELLIRSRDQSISDY..........VVMFFRFVTSGEIRKRSEFFEPFIFGLT...................NATVEQFCKSSV
A0A1E3IZJ3  GFQKDIYEEFL......DPLLVLIRSLAEGS.DATSTE......Y........SVVQALQDPERSNC..........IVVSLRLITSSFIRTHSDLFSPFLLSPAT................LLPLSTEDFCRSEV
A0A0D0CDN8  GYEKMVYEDFY......AVLEELVKNVATLD.DYGNPT......LT......DEMLLEAFQDPQTSNS..........IVVYMRMLTSAQIRLDPDNFAPFLFHPEL................GEPMEVREFCEHFV
K3YHB8      GYIEFTFEDFF......SIFVDLLESVLQGH.EAP.......IGP........EELLERTRDSQVSDY..........VVMFFRFVTSGEIQRRSEFFEPFISGLT...................NSTVAQFCKASV
K5WQI0      GYEALVYEDFY......ETLVHTIKRIINAR.PDVPKL......T.......QRGLLDAFNDTPISNS..........IMIYMRYVTSAQIKVDPFSYAPYLYNPDEPY.............ADEPMNPADFCNHFV
A0A250WPR4  HFQELVFEDAM......ELLLEQVSTIGNND.NPM.......T..K.......ETLTINMRDDMVSNM..........MVMFLRMVTSAEIQRRSDFFAPFIMGMYD.................DVSCVEVFCQRSV
A0A150GB63  GFQALVFEDAM......DVLLEQVAEISKPY.TNF.......A..H.......DKLLVDMRDDTISNL..........IVMLLRLITSCEVQRREDHFFPFILGMYD................EPPATVELFCQRHV
A8IIC5      GFQELVFEDAM......ELLLEQVKEVTKAS.DQF.......A..Q.......EKLLVNMRDDMVSNM..........IVMFLRLVTSCEVQRREDFFFPFILGMYD................EPPATVELFCQRHV
D8QXC8      GYAEFTFEDFL......AIFVEQVENALPGK.PTS.......MSIG........TLVERCRDQYISNY..........VIMFFRFVTSSEIRRRAEFFEPFILGIS...................STTVDQFCKSSV
A0A0C9VCF3  GFQEMVYEDMY......EIFTSLIHQIVTPD.VSDGHT......LT......PTILLEAMQDPQTSNS..........IVVFLRLLTSAQIRADPDNYAPFLFHQDT................FEQLPVRDFCESNV
A0A2H3ZGB8  GYVEFTFEDFF......SVFIEQLESVVQGN.QTS.......ISHE........ELLQRSRDQTISDY..........VVMFFRFVTSGEIRKRAEFFEPFIAGLT...................NSTVDQFCKASV
A0A1U7YUX3  GYAEFTFEDFF......ALFLEQLDNVLQGS.KDS.......ISHE........ELLRRSRDPSISDY..........VVMFFRFVTSGEIRKRSEFFEPFILGLT...................NASVEQFCKSSV
A0A1S4ARU0  GYAEFTFEDFF......ALFLEQLDNVLQGS.KDS.......ISHE........ELLRRSRDPSISDY..........VVMFFRFVTSGEIRKRSEFFEPFILGLT...................NASVEQFCKSSV
R9ABZ4      GFDSEIMQDFY......QPLLTILDNTKDPS.I...PL......N........EYLKVTLNDDETSNS..........IVVFLRYLTSATIRKHADEYLPFLFAYEGDLV..........VDDSGMPDIKKFCETYV
A0A0C3S2E6  GYEEAVFEDFY......EEIELLLKRILNKA.STAPPL......T.......QRGLLDAFNDAPVSNS..........ITVYMRYITAAEIKTS.PNYAPFLFNPDF................GNEMDAIEFCNCFV
A0A1Y2EUT8  GFVSIVYEDFL......ETTIELLHATD...........................LHASINDPEISNA..........AVVYIRFVTSAYIKQHAESYMPYLDEG.....................EVLPRWCERWV
A0A2H4A160  GYVEFTFEDFF......SVFIEQLESVVQGN.QTS.......ISHE........ELLQRSRDQTISDY..........VVMFFRFVTSGEIRKRAEFFEPFIAGLT...................NSTVDQFCKASV
A0A022QII4  GYAEFTFEDFF......ALFLEQLESVLSGP.EAS.......ISHE........ELVERSRDQSVSDY..........VVMFFRFITSGEIRNRSEFYEPFIQGLT...................NTSVEQFCKSSV
K4BRH8      GYAEFTFEDFF......ALFLEQLDSVLQGS.EDS.......ISHD........ELLCRSRDPSISDY..........VVMFFRFVTSGEIRKRSEFFEPFILGLT...................NTSVEQFCKSAV
A0A2G2YKF4  GYAEFTFEDFF......ALFLEQLDSVLQGS.EDS.......MSHD........ELLCRIRDPSISDY..........VVMFFRFVTSGEIRKRSEFFEPFVLGLTNASVEQLFVVPVMLELPGVNPPPYLKTQFCKSAV
A0A1Y1INL8  GYTPFTFEDFL......EIFMDQLESVLPEE.GQP.......ISL........SELEVRCRDPNVSNW..........AVMFFRFVTSAEVQRRAEFFEPFILGLT...................NLDVKKFCQTSV
S8CB89      GYAELTFEDFF......ELFLEQLEIVLVSG.DDS.......KSH........EELVMRTRDPSTSDY..........VVMFFRFITSGEIRKKSEFYEPFIQGLS...................NSSVDQFCKASV
I4YDB6      GFDSDIMLDFY......QPLLSILDNVKDPS.I.SIGD......Y.........LKVTLNDDEEVSNS..........IVVFMRYLTSATIRKNADDFIPFLFAYEGDLQ..........LDDEGMPDIKKFCETYV
D8TJ78      GFQELVFEDAM......DVLLDQVKEITKPY.TNY.......M..H.......ERLLVNMRDDTVSNL..........VVMLLRLITSCEVQRREDFFFPFIMGMYD................EPPATVELFCQRHV
A0A1S4C2D4  GYAEFTFEDFF......ALFLEQLDSVLQGS.EDS.......ISHD........ELLRRSRDPSISDY..........VVMFFRFVTSGEIRKRSEFFEPFILGLT...................NASVEQFCKSSV
A0A0D7BNF9  GFQEIVYQDFY......EELESLLRVLRPN.EITHQR......LD.....TVTLLQSFQTPEVSNS..........IVVYLRLLTSAQVRADPDAFAPFLFHPEI................GEPLEVREFCEAFI
```

```
                             α10          β3                                             β4
B8B951          TT        ~~~~~~~~~~~   ------>                               TT       -....->                   TT
               180            190       200                                  210           220                  230
B8B951       EPMGEESD.....HVHIIALSDALGVPIRVMYLD.....................RSS......CDAGNISVNHH....DFSPEANS...........SDGAA...........AAEKPYI...................
A0A1U8HSE4   EPMGEESD.....HVHITALSDALGVPIRVVYLD.....................RSS......CDIGGVSVNHH....DFLPTSG..............DKSNAKGG....STVPVKPFI...................
A0A067ST78   QAMGKEAD.....NVEIEALCRALQLNVELAYLN.....G................V..........RTDGVDFI....KFEN..................DSNN...........SAPPL...................
A0A1Y1UT00   EPCGKEAD.....HAQIMALSEALHMGVRIAYLD.....R................S..........EVQDINWV....SFG.....................PE...........TEDPL...................
W1PDW8       EPMGEESD.....HVQIIALSDALGVPIRVVYLD.....................NSS......NGPSKLDVNHH....DFIPSS.................TGS...........SSPNV...................
A0A1S2X9K8   EPMGEESD.....HVHITSLSDALGIPIRVVYLD.....................RSS......CDTGGVSVNHH....DFIPVATDLP........NASGSTEK...........NNPFI...................
A0A2I4FBU8   EPMGEESD.....HVHITALSDALGVPIRVVYLD.....................RSS......CDSGGVSVNHH....DFIPAVGDLT........NASGSSET...........VSPFI...................
S7Q8H1       EAVGKEAD.....HVQMTALARALKINFSVAYLD.....G................R.........SADGHVDFV....NFN..................DGIQD...........ELEPL...................
A0A166HFX0   EPTGKEAD.....HVQMTALSRVLQLNVKVAYLD.....G................H.........SADGKVDWV....EFQ..................CAPDS...........QAPPV...................
A0A0L9VFM7   EPMGEESD.....HVHITALSDALGVPIRVVYLD.....................RSS......CDTGGVSVNHH....DFMPVAGDLP........NASCSSVK...........NIPFI...................
M4D349       EPMGEESD.....HIHITALSDALGVAIRVVYLD.....................RSSC.....DTGGGVTVNHH....DFVPVGTTN..........EKEEASA...........AAPFI...................
A0A167IC68   EASGKEAD.....HVQEMALAKALRIKVRIAYLD.....................SS.........THGEGG.KV....DFV.EFE..............SEGEN...........GMHEI...................
A0A151VDP5   EATGKEAD.....HVQMTALARALQINIDIAYMD.....G................R..........SPD.VSSV....PLR...................GADD...........NVKPL...................
A0A287WKC8   EPMGEESD.....HVHIIALSDALGVPIRVMYLD.....................RSS......CDTGNLSVNHH....DFIPAANS............SEGD...AAMG.LNPADEKPYI...................
D7KCS8       EPMGEESD.....HIHITALSDALGVAIRVVYLD.....................RSS......CDSGGVTVNHH....DFVPVGTT............NEKDEA...........SAPFI...................
A0A165DN39   EAVGKEAD.....HPQIAALTSALTVHLKIASLD.....R................N.........VHGSEEGKV....DFVE.FQ..............NQDES...........GMEPI...................
A0A0D3H2N1   EPMGEESD.....HVHIIALSDALGVPIRVMYLD.....................RSS......CDAGNISVNHH....DFSPEANS...........SDGAA...........AAEKPYI...................
A0A177UYL2   EPVGKEAD.....HLQIAALCDFLQVSLDVAYFSRSDPAFALPDEPGAGGAGGADGS.........NQMQEVDIV....EFRIEKDAS..........ERLHGAE...........TPPHI...................
M2PGA7       EAMGKEAD.....HPQMQALAQALRVHVSVAYLD.....G................H.........SSGQNGDEV....TFVHLDK.............AVGTVP...........GAEPV...................
A0A1Y2GXH5   EAMGRESE.....EMMLIALTKVTHVSVEVAYLS.....G................N.........ENVDEVNFL....PFL..................PDTEP...........YMPPL...................
A0A1D5V3X0   EPMGEESD.....HVHIIALSDALGVPIRVMYLD.....................RSS......CDTGNLSVNHH....DFIPAANS...........SEGDA...AMG..LNPAEEKPYI...................
A0A1M2W323   EAIGKEAD.....HVQMSALSKVMKVNINVAYLD.....G................H.........DAQGTVSFV....SFQ..................NAPDP...........DTEPV...................
A0A2K2B824   EPMGEESD.....HVHITALSDALGVPIRVVYLD.....................RSS......CDAAGVSVNHH....DFIPTPRNLP.......SATGAGSES...........INPFI...................
A0A0C3BKF6   QAMGKEAD.....NVEIEALCRALQLNVDLAYLN.....G................V..........RGDAVDFI....KFRH..................DLNP...........NATPV...................
I1QLF8       EPMGEESD.....HVHIIALSDALGVPIRVMYLD.....................RSS......CDAGNISVNHH....DFSPEANS...........SDGAA...........AAEKPYI...................
A0A061E5K9   EPMGEESD.....HVHITALSDALGVPIRVVYLD.....................RSS......CDNGGVSVNHH....DFVPTSGGHS.......NATSGSTEP...........VSPFI...................
F8Q9V9       EAVGKEAD.....HVQIAALSRALKINVKIAYLD.....G................R..........DPQEVGKV....DFH.EFI..............FADDT...........TSSSL...................
A0A1S3BZ50   EPMGEESD.....HVHIIALSDALGVPIRVLYLD.....................RSS......CDSGGLSVNHH....DFVPATTEVS........SDSAASEI...........KIPFI...................
V2XBN1       EATGKEAD.....HVQMTALSRALQINIDIAYLD.....G................R.........GVNGAVEFV....KF...................QDAKT...........KLKPI...................
A0A0D7AC36   EAVGKEAD.....HVQMTALSQALGLDIDVAYLD.....G................H.........SSDGTVDFV....QFR..N...............AREDN...........SAQPL...................
A0A061E5K2   EPMGEESD.....HVHITALSDALGVPIRVVYLD.....................RSS......CDNGGVSVNHH....DFVPTSGGHS.......NATSGSTEP...........VSPFI...................
A0A2H3BLE1   EAVNKEAD.....HVQMTALSRALQLNVNVAYLD.....G................R.........NSNGLVDFV....EFR..................SAADA...........NETPL...................
I1M2E2       EPMGEESD.....HVHITALSDALGIPVRVVYLD.....................RSS......SDTGGVSVNHH....DFMPVAGDLP........NASCSSEK...........NIPFI...................
A0A067JKE5   EPMGEESD.....HVHITALSDALGVPIRVVYLD.....................RSS......CDTGGVSVNHH....DFIPMASDVP.......NPATDGSVT...........KNPFI...................
A0A1B9I0L4   EPCGKEAD.....HAQIMALAESLNIGIRIAYLD.....K................SD.......LSTSDAINWV....EFG.K.................DTTE...........QGRPL...................
A0A1S3VF63   EPMGEESD.....HVHITALSDALGVPIRVVYLD.....................RSS......CDTGGVSVNHH....DFMPVAGDLP........SASCSSIK...........NFPFI...................
A0A0C2XAB4   EGSGKEAD.....HVQITALCRILRLNVKVAYLD.....G................R...........SDTVDFV....DFQ...................NDST...........SKRFI...................
A0A2H3XG30   EPMGEESD.....HVHITALSDALGVPIRVVYLD.....................RSS......CDSGTVSINHH....DFVPSSKSPQEGT..AQSSEAKTDHP...........VTPSV...................
C5YHM5       EPMGEESD.....HVHIIALSDALGVPIRVMYLD.....................RSS......CDTGNLSVNHH....DFIPSSNASEG.....DAAMTS...........TPDAEKPYI...................
R0GRE4       EPMGEESD.....HIHITALSDALGVAIRVVYLD.....................RSS......CDNGGVTVNHH....DFVPVGT.............DEKEEA...........SAPFI...................
A0A1J7G6I2   EPMGEESD.....HVHITALSDALGVPIRVVYLD.....................RSS......CETGAVSVNHH....DFMPDDGDLS........NAVTSSEK...........KNPFI...................
B6TM99       EPMGEESD.....HVHIIALSDALGVPIRVMYLD.....................RSS......CDTGNLSVNHH....DFIPSAND...........SEGDA...ATT..PAPATEKPYI...................
A0A0A0KX37   EPMGEESD.....HVHIIALSDALGVPIRVLYLD.....................RSS......CDSGGLSVNHH....DFVPATTEVA........SGSAASEI...........KIPFI...................
R4XBU1       EAMGSEAD.....NLQINALVNALGVTVQVAHLD.....G................T........DTADGQANVV....TVVPD.................AGGT...........SIGTV...................
A0A067NID6   EAVGKEAD.....HVQMTALARTLRLNLDVAYLD.....G................R.........SADGHVDFV....EFR.....................NS...........DATPL...................
A0A2H3JM97   EAMGKEAD.....HVQIIALSRVLKVNITVAYLD.....G................R.........PHNSQEGKV....DFLE.FH..............NADIP...........GMNPV...................
M5G089       EASGKEAD.....HVQEMALARALNLCVRIAYLD.....................NSL.......GGQGAEGKVD....FVTFESE..............QEGGN...........GMHEI...................
A0A1B9J077   EPCGKEAD.....HAQIMALAEALNVGIRIAYLD.....R................SDL....GGSDADSVINWV....EFG.K.................DTSE...........QGRPL...................
M7ZPE5       EPMGEESD.....HVHIIALSDALGVPIRVMYLD.....................RSS......CDTGNLSVNHH....DFIPAANS...........SEGDA...AMG..LNPAEEKPYI...................
A0A2H3DBJ6   EAVNKEAD.....HVQMTALSRALQLNVNVAYLD.....G................R.........NSNGLVDFV....EFR..................SAPDA...........KETPL...................
A0A2K1KI58   EPMGEESD.....HVHIIALSDALGVPVRVVYLD.....................QSGD.....MNDKPVTVNKH....DFIPEGM................NTA...........VDPDV...................
A0A1Q3E3E4   DATGKEAD.....HVQMTALSRMIRTNVDVAYLD.....G................R.........GSNGQVEFV....KFQPDE...............TAEAA...........ASSPI...................
J3MUT7       EPMGEESD.....HVHIIALSDALGVPIRVMYLD.....................RSS......CDAGNISVNHH....DFIPEANS...........SEGAA...........AAEKPYI...................
OTUBL        EPMGEESD.....HIHITALSDALGVAIRVVYLD.....................RSS......CDSGGVTVNHH....DFVPVGIT...........NEKDEEA...........SAPFI...................
A0A0E0AY82   EPMGEESD.....HVHIIALSDALGVPIRVMYLD.....................RSS......CDAGNISVNHH....DFSPEANSS..........DGAAA...........AAEKPYI...................
A0A078FFK8   EPMGEESD.....HIHITALSDALGVAIRVVYLD.....................RSSC.....DTGGGVTVNHH....DFVPVGSGT.........NEKEEASS...........AAPFI...................
B9T922       EPMGEESD.....HVHITALSDALGVAIRVVYLD.....................RSS......CDTGGVSVNHH....DFIPPAGDL.........PNSN..SDGS.......LSKNPFI...................
A0A078JMQ6   ESMGEESD.....HIHITALSDALDVAIRVVYLD.....................RSS......CDSGGVTVNHH....DFVPVGTN............EKQEEA...........SAPFI...................
A0A0D2PW37   EPMGEESD.....HVHITALSDALGVPIRVVYLD.....................RSS......CDIGGVSVNHH....DFLPTSG..............DKSNAKGG....STVPVKPFI...................
B0D7Q3       EPLGKEAD.....HVEIQALCAALQLNVDVAYLN.....G................G.........SEEGVVDFI....PFRY..................ASGS...........ESLPL...................
A0A0C3MVV4   DPMGKEAD.....HVQITALSRALQVRIHIAYLD.....G................H.........SPDGKVKFV....EFDQA..................PDS...........RSEPL...................
A0A1Y2BF89   EPCGKEAD.....HAQIMALAEALNTGILVAYLD.....R................S.........DVKDGVNWV....DFG......................H...........GDDPL...................
A0A0D3BIJ7   EPMGEESD.....HIHITALSDALGVAIRVVYLD.....................RSSC.....DTGGGVTVNHH....DFVPVGSGT.........NEKEEASS...........AAPFI...................
A0A284RI41   EAVNKEAD.....HVQMTALSRALQLNVNVAYLD.....G................R.........NSNGLVDFV....EFR..................SAADA...........NETPL...................
A0A060S7F5   EAFGKEAD.....HVQINALSSMLKVNINVAYLD.....G................H.........DPNGRVSYV....KFQD.................AAEDP...........DTEPV...................
A0A2G9I7B2   EPMGEESD.....HVHITALSDALGVPIRILYLD.....................RSC......DDKGSISVNHH....DFTPAPGNP..........TNDNANA...........VKPFI...................
A0A0C3JS04   DPMGKEAD.....HVQITALSRALQVRIHIAYLD.....G................H.........SPDGKVKFV....EFDQA..................PDS...........RSEPL...................
A0A1A5ZZ56   EPCGKEAD.....HAQIMALAEALNIGIRIAYLD.....R................SD......LSSETDVINWV....EFG.K.................DTSE...........EGRPL...................
A0A165SCJ9   EAVGKEAD.....HVQMTALARALKIDFSVAYLD.....G................R.........SPDGHVDFV....KFN..................EGVDD...........GLDPL...................
A0A0K9RW17   EPMGEESD.....HVHITALSDALGVPVRVVYLD.....................NSS......CDTGGVSVNHH....DFVPCSLSN..........DGSSGLE...........INPYI...................
S8E2C4       EAVGKEAD.....HVQMTALTRALRVNIKVAYLD.....G................H.........VHGHEGNVS....FVE..FN..............DAEEA...........AIDPV...................
A0A1D6DBC7   EPMGEESD.....HVHIIALSDALGVPIRVMYLD.....................RSS......CDTGNLSVNHH....DFIPATNS...........SEGDA...AMG..LNPAEEKPYI...................
V4MQS4       EPMGEESD.....HIHITALSDALGVAIRVVYLD.....................RSS......CDNGGVTVNHH....DFVPVGT.............NEKEEA...........SAPFI...................
A0A151RBZ5   EPMGEESD.....HVHITALSDALGIPIRVVYLD.....................RSS......CDTGGVSVNHH....DFMPVAGDLP........NASCSSEK...........NIPFI...................
V7BT90       EPMGEESD.....HVHITALSDALGIPIRVVYLD.....................RSSC.....DTGGGVSVNHH....DFMPAAGDLP........TACCSSEK...........NIPFI...................
Q5KB39       EPCGKEAD.....NAQIMALAEAMNAGVRVAYLD.....R................S........EVGGKTINWV....EFG.K.................DTSE...........NARPL...................
A0A0C9XB17   EPLGKEAGKVHSYHVEIQALCAALQLNVDVAYLN.....G................G.........SEEGVVDFI....PFRY..................ASES...........ESLPL...................
A0A1R3IR70   EPMGEESD.....HVHITALSDALGVPIRVVYLD.....................RSS......CDSAGVSVNHH....DFVPTSGDHS.......NATSGSTEP...........ARPFI...................
A0A0C3FS27   ESVGKEAD.....HVQITALSRALQISVKIAYLD.....G................R.........GTDGKVDFV....DFD....................SAA...........GGDPL...................
D7TFI5       EPMGEESD.....HVHITALSDALGVPIRVVYLD.....................RST......YDIDHVIVNHH....DFIPSAGDLP.......SAGGGSSET...........TKPIV...................
A0A067DTL4   EPMGEESD.....HVHITALSDALGVPIRVVYLD.....................RSS......CDSGGASVNHH....DFIPTPGDCP.......NATSGSTET...........TIPFI...................
A0A061EBF6   EPMGEESD.....HVHITALSDALGVPIRVVYLD.....................RSS......CDNGGVSVNHH....DFVPTSGGHS.......NATSGSTEP...........VSPFI...................
A0A0H2RX12   EACGKEAD.....NVQISALTRALKLNIEIAYLD.....G................H.........SADGSVNFV....PFQNLP.................ESD...........MPNPP...................
A0A199VGJ7   EPMGEESD.....HIHITALSDALGVPIRVVYLD.....................RSS......CDAANPTVNYH....DFIPSSNPRGAISEQQDSIESSARQQ...........TKPFI...................
A0A197KHX0   EAMGRESE.....EMMLIALTKATHVSIEVAYLS.....G................N.........QAADEVNFL....PFL..................PDTAP...........YMPPL...................
A0A165QH94   EAVGKEAD.....HVQMTALTKALKVNVSVAYLD.....G................H.........VHGEEGKVD....FVQ..FK..............NVEDS...........TIDPV...................
A0A0D9XA86   EPMGEESD.....HVHIIALSDALGVPIRVMYLD.....................RSS......CDAGNISVNHH....DFIPEANS...........SEGGA...........AAEKPYI...................
G7ILC2       EPMGEESD.....HVHITALSDALGIPIRVVYLD.....................RSS......CDTGGVSVNHH....DFTPVAGDLP........SASGSSEK...........KNPFI...................
M7WQR9       EAVSREAD.....HLAITALTRALHVPLRIAYLD.....................QSLMP...YGCGGEQETGEV....NFVEFEE..............EAMKE...........GERGVE..................
A0A200QGR7   EPMGEESD.....HVHITALSDALGVPIRVMYLD.....................RSS......CDSNGVSVNHH....DFIPAVSDLQ.......GTTKSDSDL...........VKPFI...................
A0A1R3FZQ0   EPMGEESD.....HVHITALSDALGVPIRVVYLD.....................RSS......CDSAGVSVNHH....DFVPTLGDHS.......NATSGSTEP...........ARPFI...................
A0A0B2QCS2   EPMGEESD.....HVHITALSDALGIPVRVVYLD.....................RSS......SDTGGVSVNHH....DFMPVAGDLP........NASCSSEK...........NIPFI...................
J9VZ27       EPCGKEAD.....NAQIMALAEAMNAGVRVAYLD.....R................S........EVGGKTINWV....EFG.K.................DTSE...........NARPL...................
F2DNP6       EPMGEESD.....HVHIIALSDALGVPIRVMYLD.....................RSS......CDTGNLSVNHH....DFIPAANS............SEGD...AAMG.LNPADEKPYI...................
A0A0D2RIG9   EPMGEESD.....HVHITALSDALGVPIRVVYLD.....................RSS......CDIGGVSVNHH....DFLPTSG..............DKSNAKGG....STVPVKPFI...................
A0A2K1KGZ1   EPMGEESD.....HVHITALSDALGVPVRVVYLD.....................QSGD.....VNDKPVIVNNH....DFIPEGM................DAA...........VEPNV...................
I1I910       EPMGEESD.....HVHITALSDALGVPIRVMYLD.....................RSS......CDPGNLSVNHH....DFVPAANSS...........EGDAS...MTS..TPADEKPYI...................
D8QFH6       DPTGKEAD.....HVQMTALSQALGLNIDIAYLD.....G................R.........SADGTVEFV....RFR..................SAPDE...........GEKAM...................
I1ME83       EPMGEESD.....HMHITALSDALGIPIRVVYLD.....................RSS......CDTGGVSVNHH....DFMPVAGDLP........NASCSSEK...........NIPFI...................
A0A1D5V3X1   EPMGEESD.....HVHIIALSDALGVPIRVMYLD.....................RSS......CDTGNLSVNHH....DFIPAANS...........SEGDA...AMG..LNPAEEKPYI...................
A0A251R152   EPMGEESD.....HVHITALSDALGVPIRVVYLD.....................RSS......CDTGGVSVNHH....DFVPAGSDLP........NASGCSEK...........VSPFI...................
A0A0D2NU52   QAMGKEAD.....NVEIDALSHVLQLDLDLAYLN.....G................V..........RQDGVDFI....PFRN..................ASNK...........DDAPL...................
A0A0J0XBG3   EPCGKEAD.....HAQIMALAAALAVPIRVAYLD.....R................S........EVSSDVINWV....EFGGA.................AAAD...........ESRPL...................
V4RIX2       EPMGEESD.....HVHITALSDALGVPIRVVYLD.....................RSS......CDSGGASVNHH....DFIPTPGDCP.......NATSGSTET...........TIPFI...................
A0A1D6DBC6   EPMGEESD.....HVHIIALSDALGVPIRVMYLD.....................RSS......CDTGNLSVNHH....DFIPAANS...........SEGDA...AMG..LNPAEEKPYI...................
A0A287WKK4   EPMGEESD.....HVHIIALSDALGVPIRVMYLD.....................RSS......CDTGNLSVNHH....DFIPAANS............SEGD...AAMG.LNPADEKPYI...................
A0A1E3HMW3   EPCGKEAD.....HAQIMALSQALKQGIKVAYLD.....R................SE.......VGDGSVINWV....EFG.N.................DTSE...........ESRPL...................
J4H402       EAVGKEAD.....HVQITALSRALKVNINVAYLD.....G................R.........SEEGKVDFV....DFQ..................NVDEP...........GTEPV...................
A0A0W0G1T6   EATGKEAD.....HVQMTALSRALQINIDIAYLD.....G................R.........GVNGAVEFV....KF...................QDAKT...........KLKPI...................
E6RB37       EPCGKEAD.....NAQIMALAEAMNAGVRVAYLD.....R................S........EVSGKAINWV....EFG.K.................DTSE...........DARPL...................
A0A0C9SQV1   EAVGKEAD.....HVQIIALSRALEINVHIAYLD.....G................R.........DTNGRVEFV....EF....................NNAP...........GADPL...................
A0A061E3L4   EPMGEESD.....HVHITALSDALGVPIRVVYLD.....................RSS......CDNGGVSVNHH....DFVPTSGGHS.......NATSGSTEP...........VSPFI...................
A0A1U8JUW3   EPMGEESD.....HVHITALSDALGVPIRVVYLD.....................RSS......CDIGGVSVNHH....DFLPTSG..............DKSNAKGG....STVPVKPFI...................
A0A015I1G2   EAMDQEAD.....HIHVMALTKALKVPVEIAYMS.....G................S.........DAMEQVNFH....EFYPDDE.............GSKGTV...........TLKPL...................
A0A1B9FWJ4   EPCGKEAD.....HAQIMALAEALNIGVRIAYLD.....R................SDL.....GSEADSAINWV....EFG.K.................DTSE...........PGRPL...................
A0A1Y1XTK5   EAMGMESD.....QIHIIALTKVLDTPVQVVYLD.....G................S.........NNTDSVVFH....NFE.VDH..............TESAL...........SKRSL...................
W4K3W5       EAVGKEAD.....HVQVTALSRALKINVSVAYLD.....G................R.........DQGGKVDFV....EFF..................NAPEA...........GTEPL...................
A0A1U7ZAI0   EPMGEESD.....HVHITALSDALGVPIRVVYLD.....................RSS......CDTGGVIVNHH....DFLPTVSDL.........QNVA...EGG......SGTVKPFI...................
A0A0K9NYT8   EPMGAESD.....QVQIIALSDALGVPIRVVYLD.....................GS.........TTSLEVNHH....DFIPTLQDE..........KTQSN...EGSESKINQSPEPFV...................
I0YUE8       EPMGEESD.....HVHIVALTNALLVPIRVVYLD.....................RSMGAAMAGVGYDSAAVNHH....DFVPDSL.............AESAAP...........VNPRV...................
A0A251RSI4   EPMGEESD.....HVHITALCDALGVPIRVVYLD.....................RSSC.....EKEGGVSVNHH....DFVPTPADGAN......ASTSETGSE...........PNPFI...................
A0A0C9ZVK3   DPMGKEAD.....HVQIAALSRALRVRIQIAYLD.....G................R.........SSDGKVEFV....EFNHE..................PDS...........SRKPL...................
A0A177VY64   EPIGKEAD.....HLQIAALCDFLQVSLDVAYFS.....................RS...........DPAFALP....DEPGSGG..............TDGSE...........QMQAVDIVEFRIEKDASERLPGAE
A0A1Q3BVT1   EPMGEESD.....HVHITALSDALGVPIRVVYLD.....................RSS......CDAGGVSVNHH....DFIPATGNLP.......NATNGGEET...........ISPYI...................
A0A1E3IZJ3   EPCGKEAD.....HAQIMALSDALDMGVRIAYLD.....K................SD.......VEGDSVINWV....EFG.R.................DSSE...........AARPL...................
A0A0D0CDN8   DATGKEAD.....HVQMTALSRMLRINIDVAYLD.....G................R.........GSNGQVEFV....KFQPEE...............TMEVI...........VSSPI...................
K3YHB8       EPMGEESD.....HVHIIALSDALGVPIRVMYLD.....................RSS......CDTGNLSVNHH....DFIPSTNAEGP.....DAATTSTP...........ATEKPYI...................
K5WQI0       ECMGKEAD.....HVQITALTTALKVNVRVAYLD.....G................HEHG..KGPTADPDTSPINFVSFDNG....................EGE...........ELEAV...................
A0A250WPR4   EVMGEESD.....HVHIVALSDALQVPIAVVYLD.....................RSNVNR.VNEGSSEVEVETY....HFNPEP................GASV...........LRSCV...................
A0A150GB63   EPMGEESD.....HLHIVAITDALQIPVRVVYLD.....................SSGLP...TGGGSGLDATVH....DFVPDS.................CVPG...........THPRV...................
A8IIC5       EPMGEESD.....HLHIVAVTEALQIPVRVVYLD.....................SSG......LPAGGGGGGAG....ALEASCHDF.........VPDSCPPG...........TAPRV...................
D8QXC8       EPMGEESD.....HVHIIALTDALGVPVRVVYLD.....................RSIC.....DNGKTTDVNHH....DFIPGGAD................AE.......EQRRENPHV...................
A0A0C9VCF3   EAIGREAD.....HVQISALSRALKINIKVAYLD.....G................R.........GDGDKVDFV....ELSN..I..............EDGYN...........GMKEV...................
A0A2H3ZGB8   EPMGEESD.....HVHITALSDALGVPIRVMYLD.....................RSS......CDSGTLSINHH....DFVPGSNSPHEGA..AQSSEANTGHP...........VTPFV...................
A0A1U7YUX3   EPMGEESD.....HVQIIALSDALGVPIRVVYLD.....................RSS......CENNSINVNHH....DFVPTSDGM.........GNSGVSKT...........TNPSI...................
A0A1S4ARU0   EPMGEESD.....HVQIIALSDALGVPIRVVYLD.....................RSS......CENNSINVNHH....DFVPTSDGM.........GNSGVSKT...........TNPSI...................
R9ABZ4       EAFGKEAD.....HIQMTALGRVLQVDLKVCYLD.....................AS..........EKEHVDWH....ELG..VS..............GDESG...........EGNVI...................
A0A0C3S2E6   DPIGVEAD.....QVQITALTTALQVDLDVAYLDGHEHPF...............PSGI....QAQPDMSYVNV....HQFE....................NGD...........GFRPI...................
A0A1Y2EUT8   EAMDAEAD.....HLQINGLVNALHVGVDIVYLD.....R................S..........AGDEATVH....QVRP..................DEGD...........AVHVV...................
A0A2H4A160   EPMGEESD.....HVHITALSDALGVPIRVMYLD.....................RSS......CDSGTLSINHH....DFVPGSNSPHEGA..AQSSEANTGHP...........VTPFV...................
A0A022QII4   EPMGEESD.....HVHITAISDALGVAIRIVYLD.....................RSC......DDKGSVTVNHH....DFSPVARDPTNA...NINNNSNNSQP...........IKPFI...................
K4BRH8       EPMGEESD.....HVQITALSDALGVPIRVVYLD.....................RSS......CENNSINVNHH....DFIPTSREVG........NSDVSKTT...........NRPSI...................
A0A2G2YKF4   EPMSEESD.....HVQIIALSDALGVPIRVVYLD.....................RSS......CENNSINVNHH....DFIPTSSEV.........GNSDVSKT...........TNPSI...................
A0A1Y1INL8   EPMGEESD.....HVHITALTDALGIPVRIVYLD.....................RSGG.....ASDDALEINHH....DFVPESA.............PESFKP..........DTSGLL...................
S8CB89       EPMGEESD.....HIHITALSEALGVPIRVVYLD.....................RSC......DDKGNVTVNHH....DFVPASRAAGT......STTAAAAAS...........SEPLV...................
I4YDB6       EAFGKEAD.....HLQMTALGRVLDLGLKVCYLD.....................AS..........ERPTVDWH....TLN......................E...........NSKTI...................
D8TJ78       EPMGEESD.....HLHIVAITEALQIPVRVVYLD.....................SSGMP..TGGNGSGLDATMH....DFVPDS.................CVPG...........TQPRV...................
A0A1S4C2D4   EPMGEESD.....HVQIIALSDALGVPIRVVYLD.....................RSS......CENNSINVNHH....DFVPTSD..............DMGNS...........GDPSI...................
A0A0D7BNF9   EAVGKEAD.....HVQMTALSRALGLNVDVAYLD.....G................R.........DSSGQVDFV....QFR..................NPEGS...........TLTPI...................
```

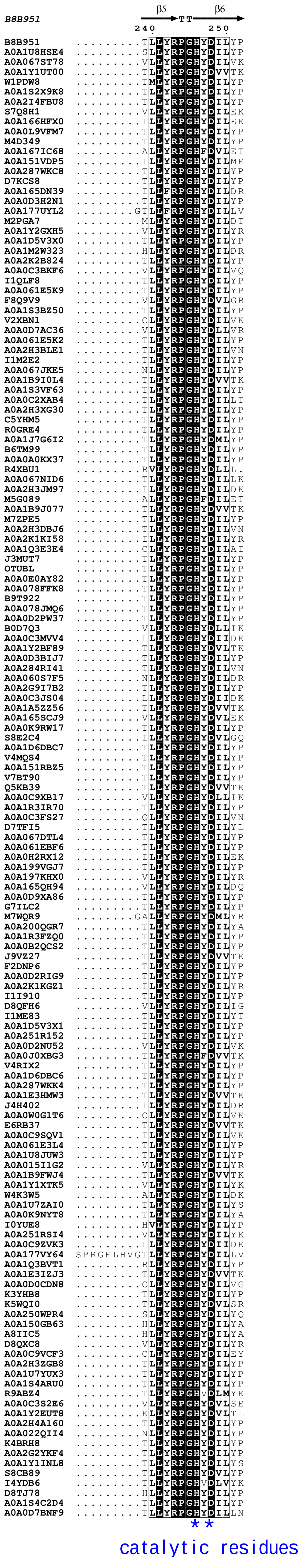
B8B951
β5
β6
TT
240
250
catalytic residues
