# Supplementary material for: Met1-specific motifs conserved in OTUB subfamily of green plants enable rice OTUB1 to hydrolyse Met1 ubiquitin chains
Source: Nat Commun. 2022 Aug 9;13:4672. doi: 10.1038/s41467-022-32364-3 (PMC9363410; doi:10.1038/s41467-022-32364-3)
Supplement: Supplementary file 7 — Reporting Summary [file 41467_2022_32364_MOESM7_ESM.pdf]

nature portfolio

Corresponding author(s): Lining LU, Ziqing MEI, Zhengguo HE, Feng WAN

Last updated by author(s): Jul 15, 2022

Reporting Summary

Nature Portfolio wishes to improve the reproducibility of the work that we publish. This form provides structure for consistency and transparency in reporting. For further information on Nature Portfolio policies, see our Editorial Policies and the Editorial Policy Checklist.

Statistics

For all statistical analyses, confirm that the following items are present in the figure legend, table legend, main text, or Methods section.

n/a	Confirmed	
☐	☒	The exact sample size (*n*) for each experimental group/condition, given as a discrete number and unit of measurement
☐	☒	A statement on whether measurements were taken from distinct samples or whether the same sample was measured repeatedly
☒	☐	The statistical test(s) used AND whether they are one- or two-sided<br>*Only common tests should be described solely by name; describe more complex techniques in the Methods section.*
☒	☐	A description of all covariates tested
☒	☐	A description of any assumptions or corrections, such as tests of normality and adjustment for multiple comparisons
☐	☒	A full description of the statistical parameters including central tendency (e.g. means) or other basic estimates (e.g. regression coefficient) AND variation (e.g. standard deviation) or associated estimates of uncertainty (e.g. confidence intervals)
☒	☐	For null hypothesis testing, the test statistic (e.g. *F*, *t*, *r*) with confidence intervals, effect sizes, degrees of freedom and *P* value noted<br>*Give P values as exact values whenever suitable.*
☒	☐	For Bayesian analysis, information on the choice of priors and Markov chain Monte Carlo settings
☒	☐	For hierarchical and complex designs, identification of the appropriate level for tests and full reporting of outcomes
☒	☐	Estimates of effect sizes (e.g. Cohen's *d*, Pearson's *r*), indicating how they were calculated

Our web collection on statistics for biologists contains articles on many of the points above.

Software and code

Policy information about availability of computer code

Data collection	Gel imaging: BIO-RAD ChemiDoc XRS+; Band intensity quantification: ImageJ software; X-ray diffraction: Shanghai Synchrotron Radiation Facility 17U, 18U and 19U; Fluorescence detection: BioTek, Synergy HT; SPR: Biacore 8K+ instrument (Cytiva); HPLC: Shimadzu LC-2030C 3D Plus with detector RID-20A using 5 µm column of Symmetry300TM C18 (Waters).
Data analysis	Band quantification: Image J; Structure visualization: PyMol 1.5.0.4; Model building and refinement: CCP4 7.0.024, COOT 0.8.9.2, Phenix.refine 1.18.2; Steady state affinity model: Biacore Insight Evaluation 3.0.12.15655; Reaction velocities and the Michaelis–Menten equation: Graphpad Prism 5.

For manuscripts utilizing custom algorithms or software that are central to the research but not yet described in published literature, software must be made available to editors and reviewers. We strongly encourage code deposition in a community repository (e.g. GitHub). See the Nature Portfolio guidelines for submitting code & software for further information.

Data

Policy information about availability of data

All manuscripts must include a data availability statement. This statement should provide the following information, where applicable:

- Accession codes, unique identifiers, or web links for publicly available datasets
- A description of any restrictions on data availability
- For clinical datasets or third party data, please ensure that the statement adheres to our policy

All uncropped and unprocessed scans are included in the Source Data file, which is provided with this paper. The mass spectrometry proteomics data generated in this study have been deposited to the ProteomeXchange Consortium via the PRIDE partner repository with the dataset identifiers PXD032822 and 10.6019/PXD032822 (http://www.ebi.ac.uk/pride). 6K9N [https://www.rcsb.org/structure/6K9N] (crystal structure of apo OsOTUB1); 6KBE [https://www.rcsb.org/

structure/6KBE] (crystal structure of OsOTUB1~Met1-diUb-DHA); 6K9P [https://www.rcsb.org/structure/6K9P] (crystal structure of OsOTUB1~Ub-PA); 1UBQ [https://www.rcsb.org/structure/1UBQ] (crystal structure of ubiquitin); 2ZFY [https://www.rcsb.org/structure/2ZFY] (crystal structure of apo hOTUB1); 3ZNZ [https://www.rcsb.org/structure/3ZNZ] (crystal structure of OTULIN OTU domain (C129A) in complex with Met1-diUb); 2W9N [https://www.rcsb.org/structure/2W9N] (crystal structure of extended Met1-diUb); 3AXC [https://www.rcsb.org/structure/3AXC] (crystal structure of compact Met1-diUb); 4DDG [https://www.rcsb.org/structure/4DDG] (crystal structure of Ubdistal~UbcH5bC85S~hOTUB1Δ1-24-Ubprox); Sequences of C65 peptidase (updated to January 2020): retrieved from database Pfam (PF10275) and supplied by this work named 'Supplementary Data 1'.

Field-specific reporting

Please select the one below that is the best fit for your research. If you are not sure, read the appropriate sections before making your selection.

- [x] Life sciences
- [ ] Behavioural & social sciences
- [ ] Ecological, evolutionary & environmental sciences

For a reference copy of the document with all sections, see nature.com/documents/nr-reporting-summary-flat.pdf

Life sciences study design

All studies must disclose on these points even when the disclosure is negative.

Sample size	3 biological replicates was chosen for all assays. The only exception was the kinetics assay for OsOTUB1-cat against Met1-diUb variants where an initial study using n=2 was used due to sample limitations.
Data exclusions	No data were excluded.
Replication	Each experiment was reproduced with multiple biological and technical replicates, with numerous controls. All attempts at replication were successful.
Randomization	No grouped samples.
Blinding	No grouped samples.

Reporting for specific materials, systems and methods

We require information from authors about some types of materials, experimental systems and methods used in many studies. Here, indicate whether each material, system or method listed is relevant to your study. If you are not sure if a list item applies to your research, read the appropriate section before selecting a response.

Materials & experimental systems

n/a	Involved in the study	
☐	☒	Antibodies
☒	☐	Eukaryotic cell lines
☒	☐	Palaeontology and archaeology
☒	☐	Animals and other organisms
☒	☐	Human research participants
☒	☐	Clinical data
☒	☐	Dual use research of concern

Methods

n/a	Involved in the study	
☒	☐	ChIP-seq
☒	☐	Flow cytometry
☒	☐	MRI-based neuroimaging

Antibodies

Antibodies used

The primary antibody: anti-GFP, anti-HSP82(rice), anti-Ubiquitin, anti-Ubiquitin-linear, anti-Ubiquitin-Lys48, anti-OsOTUB1;
The secondary antibody: Peroxidase-AffiniPure Goat Anti-Rabbit IgG, Peroxidase-AffiniPure Goat Anti-Mouse IgG.

Validation

All antibodies, except for anti-OsOTUB1, used for western blotting were purchased from commercial vendors. Validation of antibodies used in current study is described in technical data sheets provided by manufacturers websites:

anti-OsOTUB1 (BGI): antiserum was generated by injecting purified OsOTUB1 into rabbits
anti-GFP (Roche, catalog #11814460001): https://www.sigmaaldrich.cn/CN/zh/product/roche/11814460001
anti-HSP82 (rice) (Beijing Protein Innovation, catalog #AbM51099-31-PU): http://www.proteomics.org.cn/product/202.html
anti-ubiquitin (Abcam, catalog #ab134953): https://www.abcam.com/ubiquitin-antibody-epr8830-ab134953.html
anti-Met1 ubiquitin chains (Merck-millipore, catalog #MABS199): https://www.sigmaaldrich.cn/CN/zh/product/mm/mabs199
anti-Lys48 ubiquitin chains (Abcam, catalog #ab140601): https://www.abcam.com/ubiquitin-linkage-specific-k48-antibody-ep8589-ab140601.html
Peroxidase-AffiniPure Goat Anti-Rabbit IgG (Jackson 111-035-003): https://www.jacksonimmuno.com/catalog/products/111-035-003
Peroxidase-AffiniPure Goat Anti-Mouse IgG (Jackson 115-035-003): https://www.jacksonimmuno.com/catalog/products/115-035-003.
